# T cell-mediated microglial activation triggers myelin pathology in a mouse model of amyloidosis

**DOI:** 10.1038/s41593-024-01682-8

**Published:** 2024-06-27

**Authors:** Shreeya Kedia, Hao Ji, Ruoqing Feng, Peter Androvic, Lena Spieth, Lu Liu, Jonas Franz, Hanna Zdiarstek, Katrin Perez Anderson, Cem Kaboglu, Qian Liu, Nicola Mattugini, Fatma Cherif, Danilo Prtvar, Ludovico Cantuti-Castelvetri, Arthur Liesz, Martina Schifferer, Christine Stadelmann, Sabina Tahirovic, Ozgun Gokce, Mikael Simons

**Affiliations:** 1 Institute of Neuronal Cell Biology, Technical University Munich, Munich, Germany; 2 German Center for Neurodegenerative Diseases (DZNE), Munich, Germany; 3 Institute for Stroke and Dementia Research, University Hospital of Munich, LMU Munich, Munich, Germany; 4 Department of Neurodegenerative Diseases and Geriatric Psychiatry, University Hospital Bonn, Bonn, Germany; 5 Department of Neuropathology, University Medical Center Göttingen, Göttingen, Germany; 6 Munich Cluster of Systems Neurology (SyNergy), Munich, Germany; 7 German Center for Neurodegenerative Diseases (DZNE), Bonn, Germany

**Keywords:** Glial biology, Experimental models of disease

## Abstract

Age-related myelin damage induces inflammatory responses, yet its involvement in Alzheimer’s disease remains uncertain, despite age being a major risk factor. Using a mouse model of Alzheimer’s disease, we found that amyloidosis itself triggers age-related oligodendrocyte and myelin damage. Mechanistically, CD8^+^ T cells promote the progressive accumulation of abnormally interferon-activated microglia that display myelin-damaging activity. Thus, our data suggest that immune responses against myelinating oligodendrocytes may contribute to neurodegenerative diseases with amyloidosis.

## Abstract (web summary)

The involvement of age-related myelin damage in Alzheimer’s disease is unclear. Kedia et al. show that T cell-mediated microglia activation triggers a response against myelinating oligodendrocytes contributing to neurodegenerative diseases with amyloidosis.

## Main

Age-related brain damage presents an important risk for several neurological conditions, but it is unknown which oligodendrocyte and myelin alterations contribute to the development of these diseases. A key feature of age-related brain injury are focal white matter (WM) pathologies, which are linked to an elevated likelihood of dementia including Alzheimer’s disease (AD)^1^. Aging is associated with distinct oligodendrocyte responses, consisting of disease-associated Serpina3n^+^C4b^+^ and interferon (IFN)-responsive STAT1^+^B2M^+^ oligodendrocyte states^2^, which are also detected together with myelin alterations in AD and its mouse models^3–12^. Thus, we asked whether age-related oligodendrocyte and myelin alterations contribute to the pathology of AD.

**Fig. 1:**
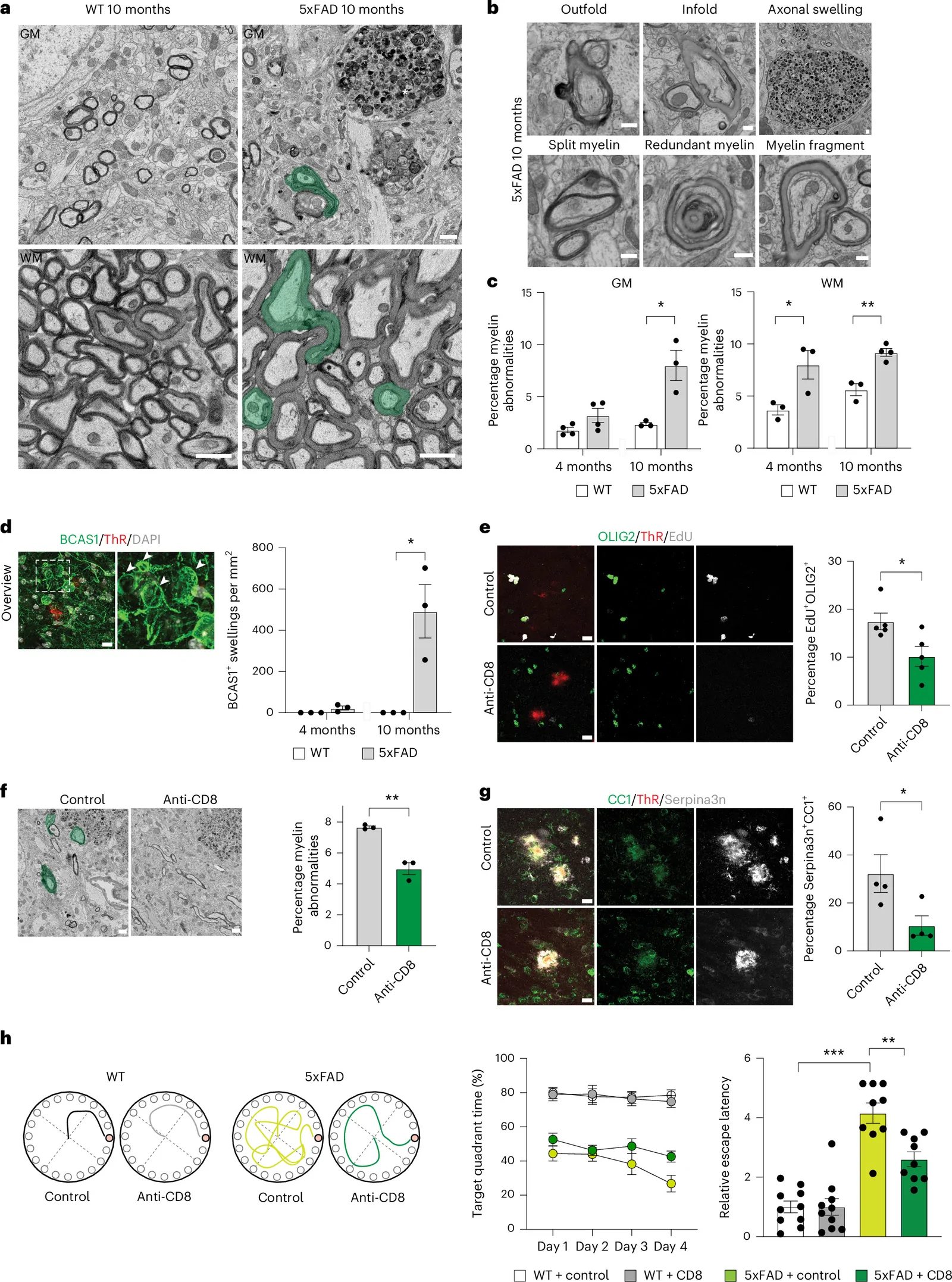
Oligodendrocyte and myelin damage is ameliorated by CD8^+^ T cell depletion in 5xFAD mice. **a**, SEM images showing myelin abnormalities (green) in WT and 5xFAD mice aged 10 months. The asterisk shows an axonal swelling with thin myelin. **b**,**c**, Images (**b**) and quantification (**c**) of different myelin abnormalities in 5xFAD brain sections. Unpaired two-sided Student’s *t*-test for *n* = 3–4 animals (GM, WT 10 months, 5xFAD 10 months, *P = 0.018; WM, WT 4 months, 5xFAD 4 months, *P = 0.041; WM, WT 10 months, 5xFAD 10 months, **P = 0.002). **d**, Image of BCAS1^+^ (green) swellings (shown with white arrowheads) lacking a nucleus (stained with DAPI) in 5xFAD mice aged 10 months. Thiazine red (ThR) indicates plaques. Quantifications of BCAS1^+^ swellings in 4-month old and 10-month old WT and 5xFAD mice. Unpaired two-sided Student’s *t*-test for *n* = 3 animals (WT 10 m, 5xFAD 10 m, *P = 0.019). **e**, Image and quantification showing the percentage of EdU^+^ (gray) OLIG2^+^ (green) cells in the cortex of control or CD8^+^ T cell-depleted 5xFAD mice. ThR (red) indicates plaques; unpaired two-sided Student’s *t*-test for *n* = 5 animals (control, anti-CD8, *P = 0.027). **f**, SEM images and quantification of myelinated axons with myelin abnormalities (green) in the cortex of control or CD8^+^ T cell-depleted 5xFAD mice; unpaired Student’s *t*-test for *n* = 3 animals (control, anti-CD8, **P = 0.002). **g**, Image and quantification showing the percentage of Serpina3n^+^ (gray) CC1^+^ (green) oligodendrocytes in the cortex of control or CD8^+^ T cell-depleted 5xFAD mice. ThR (red) indicates plaques. Unpaired two-sided Student’s *t*-test for *n* = 4 animals (control, anti-CD8, *P = 0.049). **h**, Behavior of control or CD8^+^ T cell-depleted WT and 5xFAD mice in the Barnes maze. Left: Tracing graphs in platform trials in the Barnes maze. Right: Quantification of the target quadrant time (%) on the platform during the training sessions and relative escape latency to the target hole in the probe trial. One-way analysis of variance (ANOVA) with Tukey’s post hoc analysis for *n* = 9–10 animals (WT control, 5xFAD control, ***P = 0.0000000069; 5xFAD control, 5xFAD anti-CD8, **P = 0.0019). Each point on the graph represents one animal. Data are presented as the mean ± s.e.m. **a**,**f**, Scale bar, 1 µm. **b**, Scale bar, 0.4 µm. **d**,**e**,**g**, Scale bar, 10 µm. Source data

**Extended Data Fig. 1:**
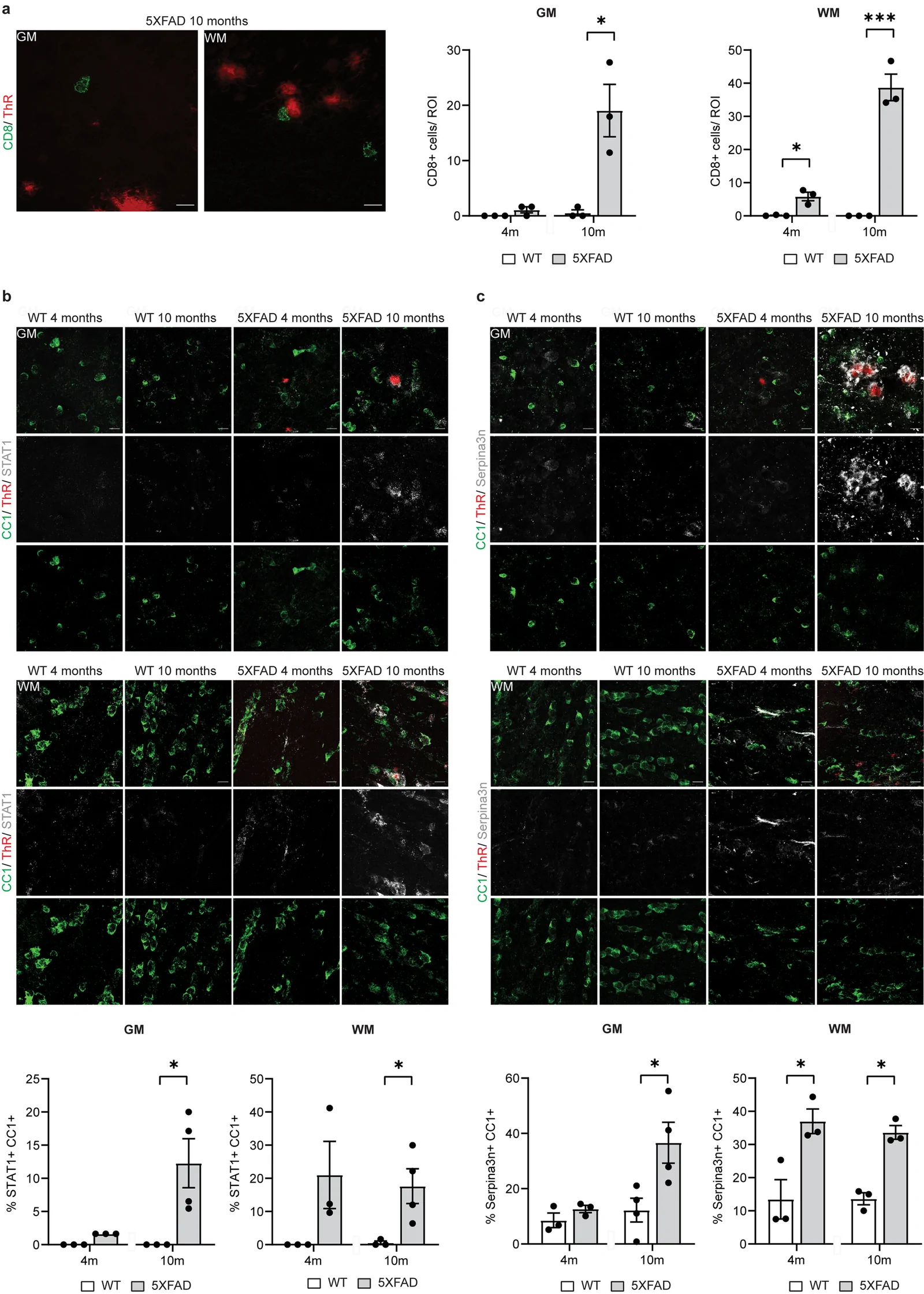
Characterization of CD8^+^ T cells and disease-associated and interferon-responsive oligodendrocyte states in *5xFAD* mice. **a**, Representative image showing CD8^+^ T-cells (green) in the cortex and corpus callosum of *5xFAD* mice aged 10 months. Plaques are stained with Thiazine Red (ThR, red). Scale bar, 10 µm. Quantifications indicate the number of CD8^+^ T cells. Statistical significance was determined for n = 3 animals using unpaired two-sided Student’s t-test (GM, WT 10 m, *5xFAD* 10 m, *p = 0.017; WM, WT 4 m, 5XFAD 4 m, *p = 0.011; WM, WT 10 m, *5xFAD* 10 m, ***p = 0.0006). **b**, Representative image showing STAT1^+^ (grey) CC1^+^ oligodendrocytes (green) in the cortex (upper panel) and corpus callosum (lower panel) of WT and *5xFAD* mice aged 4 and10 months. Plaques are stained with Thiazine Red (ThR, red). Scale bar, 10 µm. Quantifications indicate the percentage of CC1^+^ oligodendrocytes also positive for STAT1. Statistical significance was determined for n = 3-4 animals using unpaired two-sided Student’s t-test (GM, WT 10 m, *5xFAD* 10 m, *p = 0.037; WM, WT 10 m, *5xFAD* 10 m, *p = 0.040). **c**, Representative image showing Serpina3n^+^ (grey) CC1^+^ oligodendrocytes (green) in the cortex (upper panel) and corpus callosum (lower panel) of WT and *5xFAD* mice aged 4 and10 months. Plaques are stained with Thiazine Red (ThR, red). Scale bar 10 µm. Quantifications indicate the percentage of CC1^+^ oligodendrocytes also positive for Serpina3n. Statistical significance was determined for n = 3-4 animals using unpaired two-sided Student’s t-test (GM, WT 10 m, *5xFAD* 10 m, *p = 0.029; WM, WT 4 m, 5XFAD 4 m, *p = 0.028; WM, WT 10 m, *5xFAD* 10 m, *p = 0.001). Each point on the graph represents one animal. Data is presented as mean ± SEM. Source data

**Extended Data Fig. 2:**
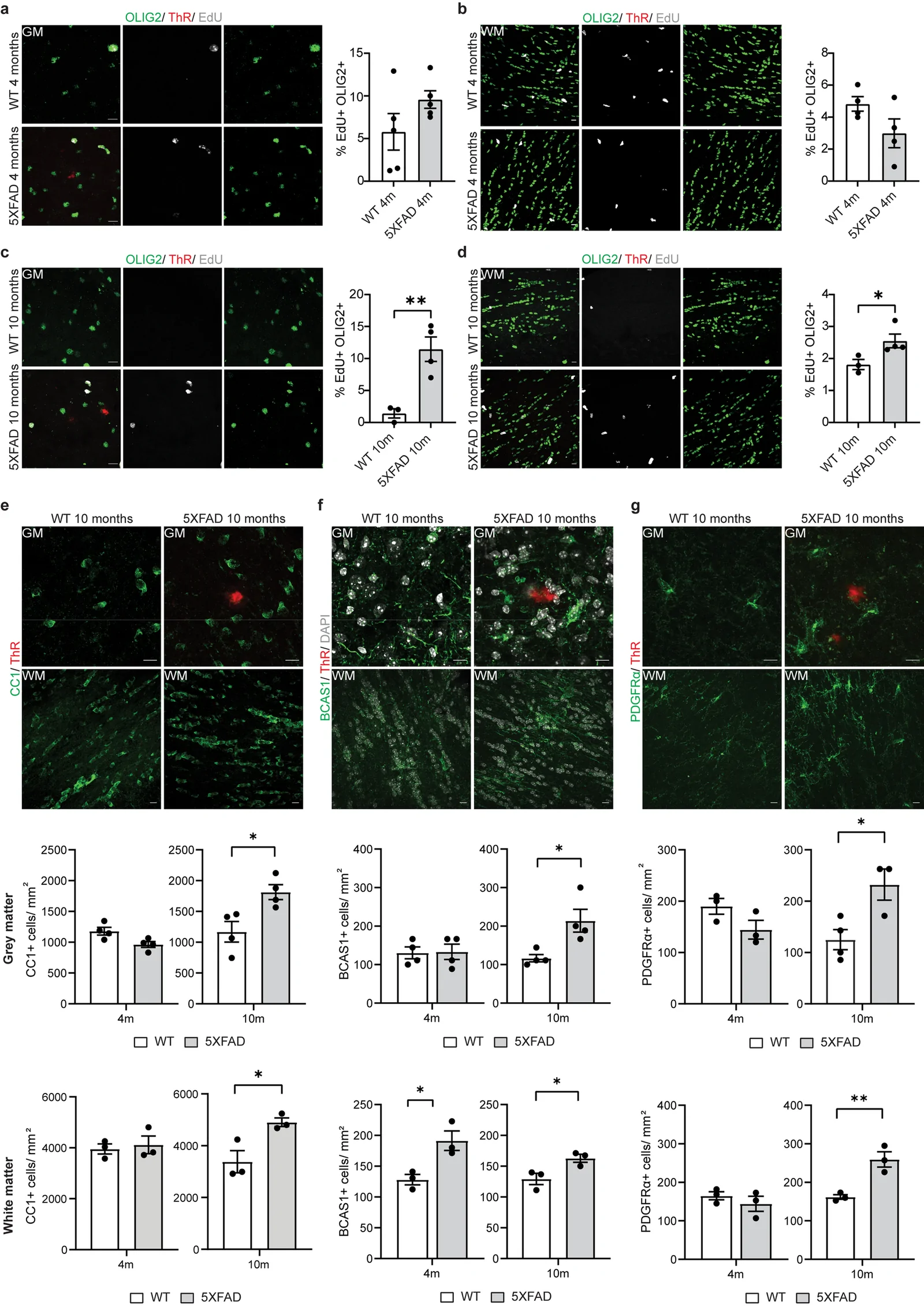
Evidence of increased oligodendrocyte proliferation in *5xFAD* mice. **a-d**, Representative image and quantification showing percentage of EdU^+^ (grey) OLIG2^+^ (green) cells in the cortex and corpus callosum of WT and *5xFAD* mice aged 4 and 10 months. Plaques are stained with Thiazine Red (ThR, red). Scale bar, 10 µm. Statistical significance was determined for n = 3-4 animals using unpaired two-sided Student’s t-test (GM, WT 10 m, *5xFAD* 10 m, **p = 0.0079; WM, WT 10 m, *5xFAD* 10 m, *p = 0.049). **e**, Representative image and quantification showing number of CC1^+^ (green) oligodendrocytes in the cortex and corpus callosum of WT and *5xFAD* mice aged 4 and 10 months. Plaques are stained with Thiazine Red (ThR, red). Scale bar 10 µm. Statistical significance was determined for n = 3-4 animals using unpaired two-sided Student’s t-test (GM, WT 10 m, *5xFAD* 10 m, *p = 0.020; WM, WT 10 m, *5xFAD* 10 m, n = 3 animals, *p = 0.029). **f**, Representative image and quantification showing number of BCAS1^+^ (green) oligodendrocytes in the cortex and corpus callosum of WT and *5xFAD* mice aged 4 and 10 months. Plaques are stained with Thiazine Red (ThR, red). Scale bar, 10 µm. Statistical significance was determined for n = 3-4 animals using unpaired two-sided Student’s t-test (GM, WT 10 m, *5xFAD* 10 m, *p = 0.020; WM, WT 4 m, *5xFAD* 4 m, *p = 0.024; WM WT 10 m, *5xFAD* 10 m, *p = 0.039). **g**, Representative image and quantification showing number of PDGFRα^+^ (green) oligodendrocyte precursor cells in the cortex and corpus callosum (lower panel) of WT and *5xFAD* mice aged 4 and 10 months. Plaques are stained with Thiazine Red (ThR, red). Scale bar 10 µm. Statistical significance was determined for n = 3-4 animals using unpaired two-sided Student’s t-test (GM, WT 10 m, *5xFAD* 10 m, *p = 0.026; WM, WT 10 m, *5xFAD* 10 m, **p = 0.009). Each point on the graph represents one animal. Data is presented as mean ± SEM. Source data

We used the 5xFAD model of amyloidosis and detected an increase in STAT1^+^CC1^+^ and Serpina3n^+^CC1^+^ oligodendrocytes, initially in WM, and at 10 months both in white and gray matter (GM) (Extended Data Fig. 1). Scanning electron microscopy (SEM) revealed abnormal myelin ultrastructure with myelin outfoldings, myelin splitting, whorls of degenerated myelin and redundant myelin (Fig. 1a–c and Supplementary Fig. 1b). In addition, there were abnormal BCAS1^+^ premyelinating oligodendrocytes with fragmented processes and large swellings, which were often positive for markers of myelin, myelin-associated glycoprotein and myelin basic protein (MBP) (Fig. 1d and Supplementary Fig. 1c,d). By analyzing consecutive sections, we observed that BCAS1^+^ cells were often without nucleus, possibly representing remnants of dying cells (Fig. 1d). These pathological features were accompanied by an increase in 5-ethynyl-2′-deoxyuridine (EdU)^+^OLIG2^+^ cells, PDGFRα^+^ oligodendrocyte progenitor cells, premyelinating BCAS1^+^ cells and mature CC1^+^ oligodendrocytes (Extended Data Fig. 2), indicating that myelin damage is associated with regenerative responses in 5xFAD mice.

**Extended Data Fig. 3:**
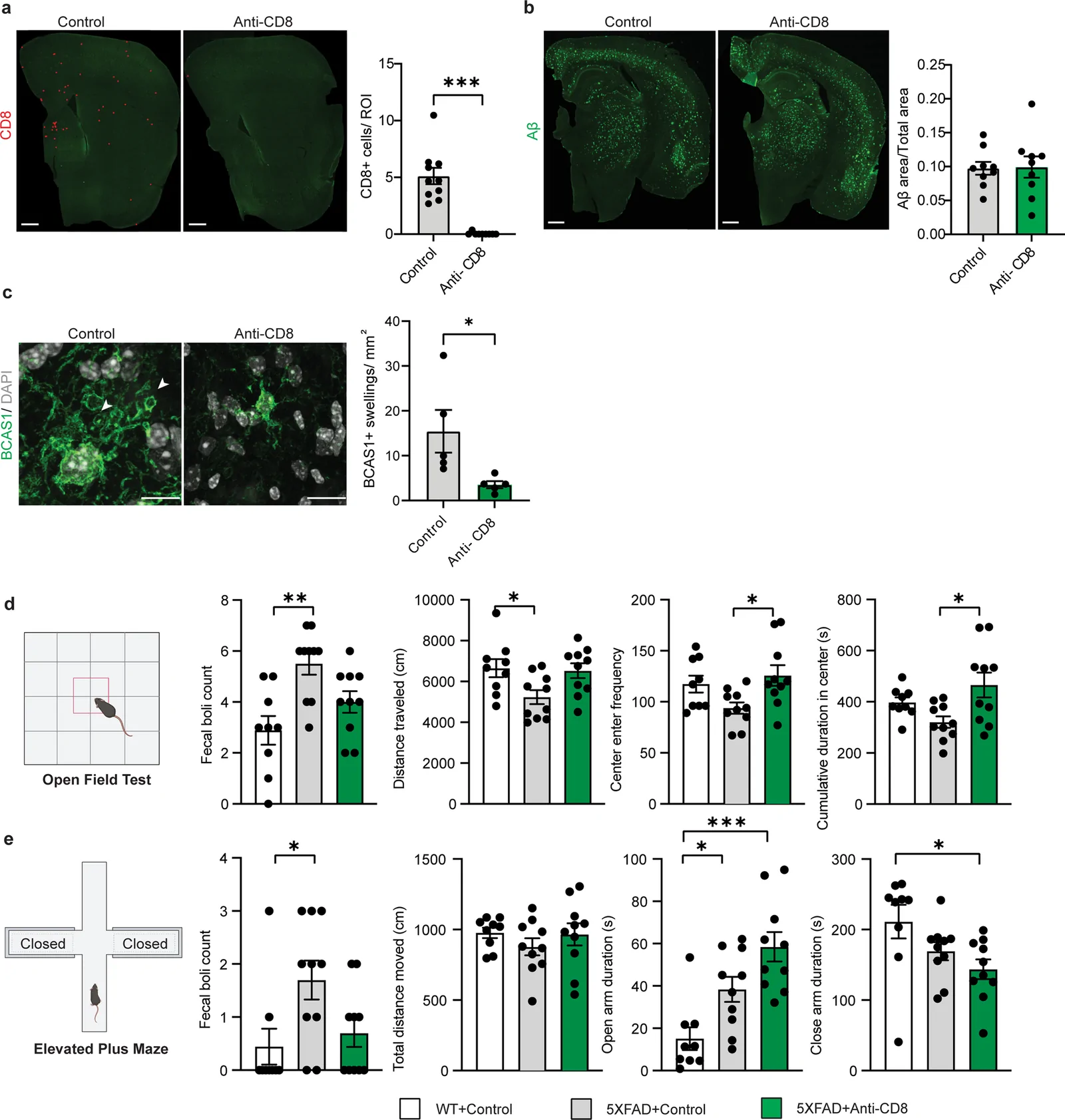
Effect of CD8^+^ depletion on behavior and on amyloid plaque load in *5xFAD* mice. **a**, Representative image and quantification showing number of CD8^+^ T cells in mice (7.5 month-old) treated with anti-CD8 and isotype control antibodies. Each dot (red) represents one CD8^+^ T cell. Scale bar, 500 µm. Statistical significance was determined for n = 9–10 animals using unpaired two-sided Student’s t-test (control, anti-CD8, ***p = 0.001). **b**, Representative image showing Aβ plaque load (green) in mice (7.5 month-old) treated with anti-CD8 and isotype control antibodies. Scale bar, 500 µm. Quantifications indicate proportion of Aβ positive area normalized to total area in the cortex. Statistical significance was determined for n = 9 animals using unpaired Student’s t-test. **c**, Representative image and quantification indicating number of BCAS1^+^ (green) swellings lacking a nucleus (stained with DAPI) in the cortex of 7.5 month-old *5xFAD* mice treated with anti-CD8 and isotype control antibodies. Scale bar, 10 µm. Statistical significance was determined for n = 5 animals using unpaired two-sided Student’s t-test (control, anti-CD8, *p = 0.038). **d**, Behavior of 7.5 month-old WT and *5xFAD* mice following 6-week treatment with anti-CD8 and isotype control antibodies in an open-field test. Quantifications indicate fecal boli count, total distance travelled, frequency to enter the center and cumulative time spent in the center. Statistical significance was determined for n = 9-10 animals by one-way ANOVA with Tukey’s post hoc test. (Fecal boli count, WT, *5xFAD*+control, **p = 0.0017; Distance traveled, WT, *5xFAD*+control,*p = 0.03; Center enter frequency, *5xFAD*+control, *5xFAD*+anti-CD8, *p = 0.02; Cumulative duration in center, *5xFAD*+control, *5xFAD*+anti-CD8, *p = 0.01). **e**, Behavior of 7.5 month-old mice following 6-week treatment with anti-CD8 and isotype control antibodies in an elevated plus maze test. Quantifications indicate fecal boli count, total distance travelled, total time spent in the open arms and total time spent in the closed arms. Statistical significance was determined for n = 9-10 animals by one-way ANOVA with Tukey’s post hoc test. (Fecal boli count, WT, *5xFAD*+control, *p = 0.03; Open arm duration, WT, *5xFAD*+control, *p = 0.03; Open arm duration, WT, *5xFAD*+anti-CD8, ***p = 0.0001; Close arm duration, WT, *5xFAD*+anti-CD8, *p = 0.02). Each point on the graph represents one animal. Data is presented as mean ± SEM. Source data

**Extended Data Fig. 4:**
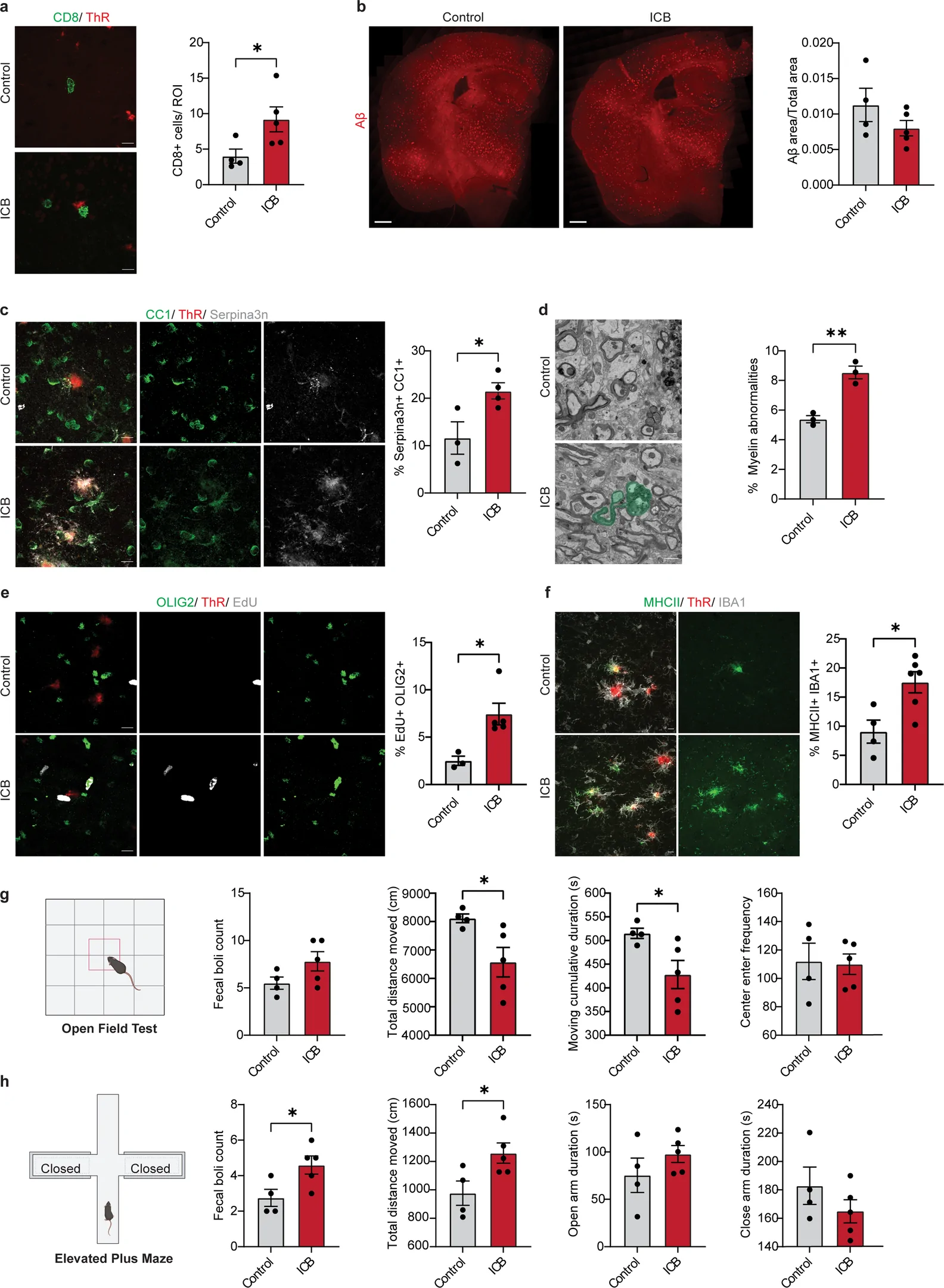
Treatment with immune checkpoint blockage exacerbates oligodendrocyte and myelin damage in *5xFAD* mice. **a**, Image and quantification showing number of CD8^+^ T cells (green) in the cortex of control or anti-PD1 and CTLA4 (ICB) treated *5xFAD* mice. Plaques are stained with Thiazine Red (ThR, red). Scale bar, 10 µm. Statistical significance was determined for n = 4-5 animals using unpaired two-sided Student’s t-test (control, ICB, *p = 0.048). **b**, Image and quantification showing Aβ plaque load (red) normalized to total area in the cortex for control or anti-PD1 and CTLA4 (ICB) treated *5xFAD* mice. Scale bar, 500 µm. Statistical significance was determined for n = 4-5 animals using unpaired Student’s t-test. **c**, Representative image and quantification showing percentage of Serpina3n^+^ (grey) CC1^+^ (green) oligodendrocytes in the cortex of control or anti-PD1 and CTLA4 (ICB) treated *5xFAD* mice. Plaques are stained with Thiazine Red (ThR, red). Scale bar, 10 µm. Statistical significance was determined for n = 3-4 animals using unpaired two-sided Student’s t-test (control, ICB, *p = 0.036). **d**, SEM images and quantification of myelinated axons with myelin abnormalities (green) in the cortex of control or anti-PD1 and CTLA4 (ICB) treated *5xFAD* mice. Statistical significance was determined for n = 3 animals using unpaired two-sided Student’s t-test (control, ICB, *p = 0.003). **e**, Image and quantification showing percentage of EdU^+^ (grey) OLIG2^+^ (green) cells in the cortex of control or anti-PD1 and CTLA4 (ICB) treated *5xFAD* mice. Plaques are stained with Thiazine Red (ThR, red). Scale bar, 10 µm. Statistical significance was determined for n = 3-5 animals using unpaired two-sided Student’s t-test (control, ICB, *p = 0.019). **f**, Image and quantification indicating percentage of MHCII^+^ (green) IBA1^+^ (grey) cells in the cortex of control or anti-PD1 and CTLA4 (ICB) treated *5xFAD* mice. Plaques are stained with Thiazine Red (ThR, red). Scale bar, 10 µm. Statistical significance was determined for n = 4-6 animals using unpaired two-sided Student’s t-test (control, ICB, *p = 0.015). **g**, Behavior of control or anti-PD1 and CTLA4 (ICB) treated *5xFAD* mice in an open-field test. Quantifications indicate fecal boli count, total distance travelled, frequency to enter the center and cumulative time spent in the center. Statistical significance was determined for n = 4-5 animals using unpaired Student’s t-test (Distance traveled, *p = 0.037; Center enter frequency, *p = 0.041; Cumulative duration in center, *p = 0.014). **h**, Behavior of control or anti-PD1 and CTLA4 (ICB) treated *5xFAD* mice in an elevated plus maze test. Quantifications indicate fecal boli count, total distance travelled, total time spent in the open arms and closed arms. Statistical significance was determined for n = 4-5 animals using unpaired Student’s t-test (Fecal boli count, *p = 0.036; Distance traveled, *p = 0.037). Each point on the graph represents one animal. Data is presented as mean ± SEM. Source data

We examined the inflammatory reactions in the WM and GM, and detected a small but significantly elevated number of CD8^+^ T cells at 10 months (Extended Data Fig. 1). To determine whether CD8^+^ T cells contribute to oligodendrocyte and myelin pathology in 5xFAD mice, we performed antibody-mediated CD8^+^ T cell depletion for 6 weeks starting in 6-month-old 5xFAD mice, a time point when CD8^+^ T cells were starting to appear in the GM. The treatment did not lead to significant changes in the amyloid plaque load (Extended Data Fig. 3b). However, the number of Serpina3n^+^ oligodendrocytes and BCAS1^+^ swellings were reduced (Fig. 1g and Extended Data Fig. 3c). In addition, the EdU labeling experiments revealed a decrease in the number of EdU^+^OLIG2^+^ cells (Fig. 1e); EM showed diminished myelin abnormalities in the cortex of CD8^+^ T cell-depleted 5xFAD mice (Fig. 1f). To assess behavioral deficits, we conducted the Barnes maze test and observed that CD8^+^ T cell-depleted 5xFAD mice exhibited a significantly lower relative escape latency during the probe trial (Fig. 1h), pointing to improved spatial learning and memory performance. Additionally, CD8^+^ T cell depletion partially reversed anxiety-related behaviors in 5xFAD mice (Extended Data Fig. 3d,e). Subsequently, we conducted the inverse experiment by administering immune checkpoint blockage therapy to mice. Treatment of 6-month-old mice with anti-programmed cell death protein (PD-1) and anti-cytotoxic T lymphocyte-associated protein 4 (CTLA-4) antibodies for 6 weeks resulted in an increase in the number of CD8^+^ T cells (Extended Data Fig. 4a). The treatment did not lead to significant changes in the amyloid plaque load (Extended Data Fig. 4b). Instead, we found an increase in the number of Serpina3n^+^ oligodendrocytes and EdU^+^OLIG2^+^ cells (Extended Data Fig. 4c,e), and using EM, an increase in the number of myelin abnormalities (Extended Data Fig. 4d). Opposite to CD8^+^ T cell elimination, anxiety-related behaviors were further exacerbated after checkpoint inhibitor treatment in 5xFAD mice (Extended Data Fig. 4g,h). Together, these data provide evidence for a role of CD8^+^ T cells in amplifying oligodendrocyte and myelin damage in 5xFAD mice.

**Extended Data Fig. 5:**
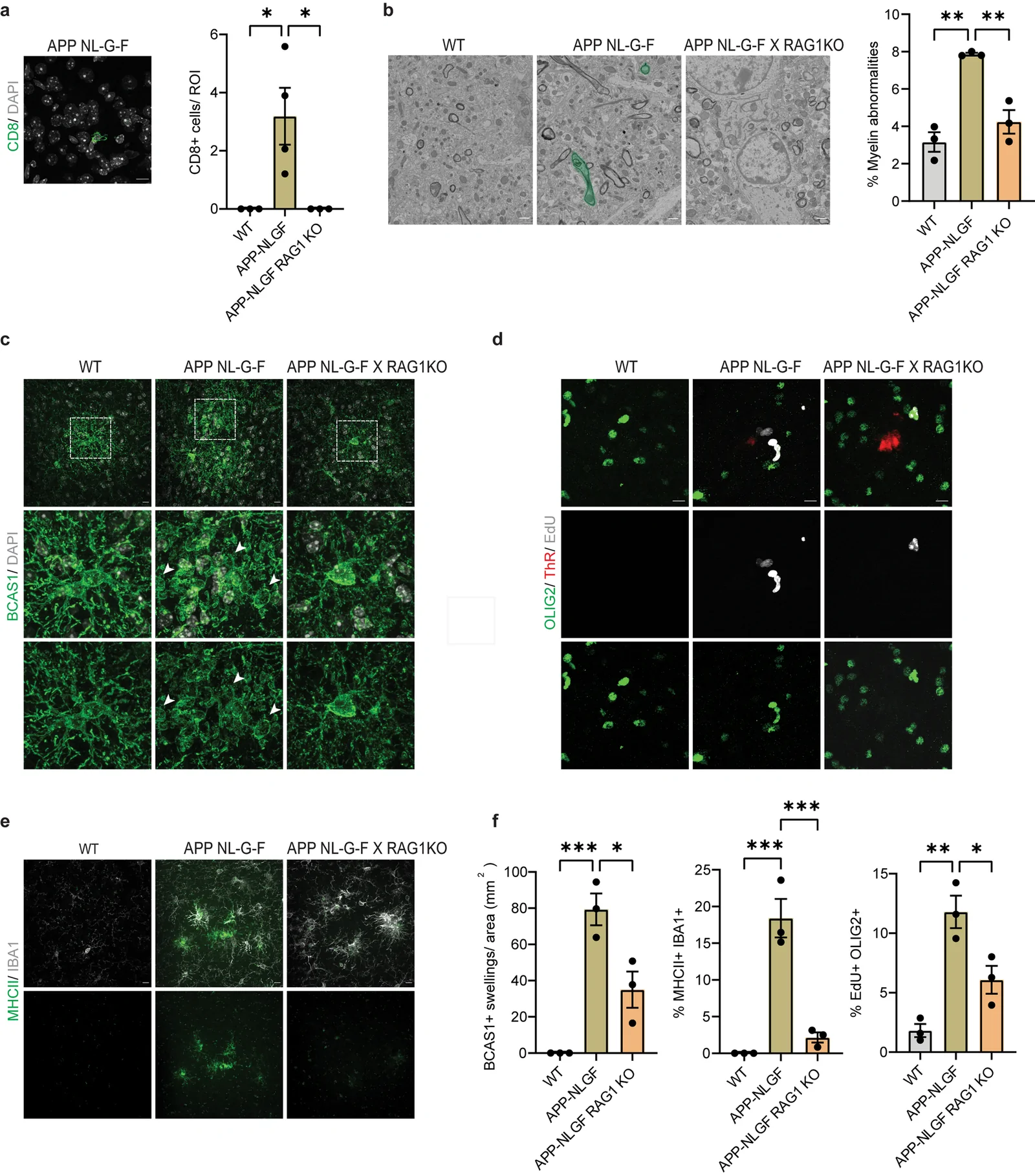
Absence of functional lymphocytes reduces oligodendrocyte and myelin damage in *APP-NLGF* mice. **a**, Representative image showing CD8^+^ T cells (green) in the cortex of *APP-NLGF* mice. Scale bar, 10 µm. Quantifications indicate the number of CD8^+^ T cells in the cortex of WT, *APP-NLGF* and *APP-NLGF* X *RAG1KO* mice aged 12 months. Statistical significance was determined for n = 3-4 animals by one-way ANOVA with Tukey’s post hoc test (WT, *APP-NLGF*, *p = 0.0326; *APP-NLGF*, *APP-NLGF* X *RAG1KO*, *p = 0.032). **b**, Representative scanning electron microscopy images of the cortex of WT, *APP-NLGF* and *APP-NLGF* X *RAG1KO* mice aged 12 months. Myelin abnormalities are shown in green. Scale bar, 1 µm. Quantifications indicate total percentage of myelinated axons with myelin abnormalities. Statistical significance was determined for n = 3 animals by one-way ANOVA with Tukey’s post hoc test (WT, *APP-NLGF*, **p = 0.001; *APP-NLGF*, *APP-NLGF* X *RAG1KO*, **p = 0.004). **c**, Representative image of BCAS1^+^ (green) swellings lacking a nucleus (stained with DAPI) in the cortex of WT, *APP-NLGF* and *APP-NLGF* X *RAG1KO* mice aged 12 months. Scale bar, 10 µm. **d**, Representative image showing EdU^+^ (grey) OLIG2^+^ (green) cells in the cortex of WT, *APP-NLGF* and *APP-NLGF* X *RAG1KO* mice aged 12 months. Plaques are stained with Thiazine Red (ThR, red). Scale bar, 10 µm. **e**, Representative image showing MHCII^+^ (green) IBA1^+^ (grey) cells in the cortex of WT, *APP-NLGF* and *APP-NLGF* X *RAG1KO* mice aged 12 months. Scale bar, 10 µm. **f**, Quantifications indicate the number of BCAS1 swellings (WT, *APP-NLGF*, ***p = 0.0008; *APP-NLGF*, *APP-NLGF* X *RAG1KO*, *p = 0.0154), percentage of IBA1^+^ cells positive for MHCII (WT, *APP-NLGF*, ***p = 0.0004; *APP-NLGF*, *APP-NLGF* X *RAG1KO*, ***p = 0.0008), percentage of OLIG2^+^ cells also positive for EdU (WT, *APP-NLGF*, **p = 0.0015; *APP-NLGF*, *APP-NLGF* X *RAG1KO*, *p = 0.0230). Statistical significance was determined for n = 3 animals by one-way ANOVA with Tukey’s post hoc test. Each point on the graph represents one animal. Data is presented as mean ± SEM. Source data

To ascertain whether these effects were reproducible in another AD mouse model, we used knock-in mice carrying multiple mutations in the *APP* gene (*APP*^*NLGF*^) and observed a significant increase in CD8^+^ T cells, EdU^+^OLIG2^+^ cells, Serpina3n^+^ oligodendrocytes and BCAS1^+^ cells with swellings, and using EM more myelin abnormalities compared to control mice at 12 months of age (Extended Data Fig. 5). On crossing *APP*^*NLGF*^ with *Rag1* knockout (KO) mice (*Rag1* KO), which lack functional lymphocytes, we noted a substantial reduction in all these responses (Extended Data Fig. 5), supporting our conclusion that lymphocytes have a role in driving oligodendrocyte alterations.

**Fig. 2:**
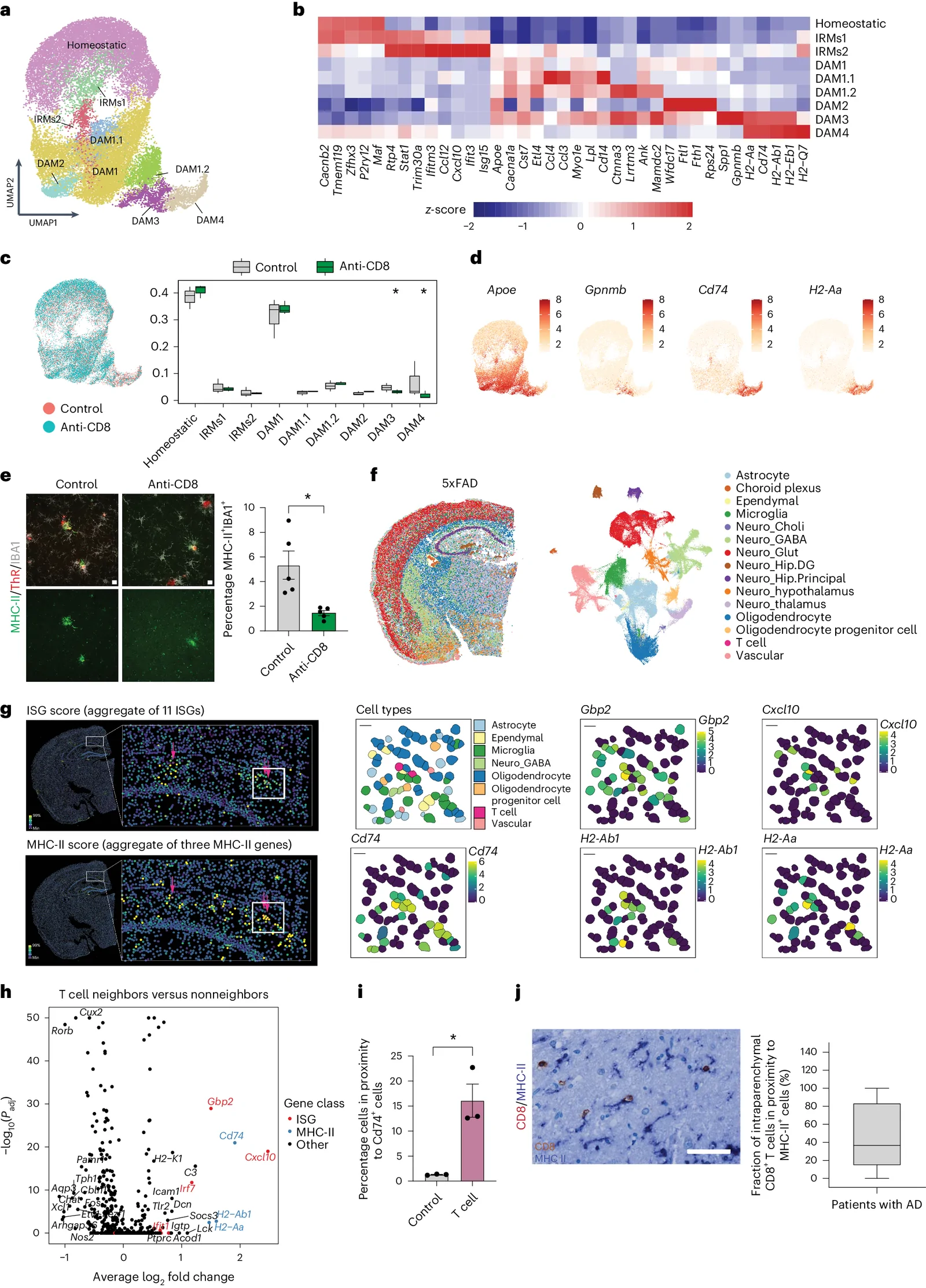
CD8^+^-mediated microglial activation in 5xFAD mice. **a**, UMAP plot of microglia, colored according to the identified populations. **b**, Heatmaps of scaled average expression of selected marker genes of microglial populations. **c**, Left: UMAP plots of microglia. Right: Proportions of different microglial populations in control (gray) or CD8^+^ T cell-depleted (green) 5xFAD mice. The central line represents median, the boxes the upper and lower quartiles of the proportions, and the whiskers show the full range of all populations. Asterisks indicate statistical significance for *n* = 3 animals (using scCODA). Statistical significance was determined for *n* = 3 animals using scCODA. **d**, UMAP plots of microglia colored according to the expression of selected gene markers. **e**, Image and quantification showing the percentage of MHC-II^+^ (green) IBA1^+^ (gray) cells in the cortex of control or CD8^+^ T cell-depleted 5xFAD mice. ThR (red) indicates plaques. Unpaired two-sided Student’s *t*-test for *n* = 5 animals (control, anti-CD8, *P = 0.010). Data are presented as the mean ± s.e.m. **f**, UMAP embedding and spatial plots of single-cell transcriptional profiles measured using MERFISH. Cells are colored according to the major cell type. *n* = 306,537 segmented single cells from five brain sections from *n* = 3, 9.5-month-old 5xFAD animals. **g**, Left: MERFISH spatial plot showing the values of ISG (top) and the MHC-II (bottom) expression score. The arrows in the zoom-in show the positions of T cells. Right: Plots show the cell type identities and expression of selected ISG and MHC-II genes. **h**, Volcano plot of differential gene expression between 50 nearest neighbors of T cells and remaining cells. Bonferroni-adjusted *P* values from a Wilcoxon rank-sum test are shown. **i**, Quantification of *Cd74*^+^ neighboring cells in proximity to control and T cells. Unpaired two-sided Student’s *t*-test (control, T cell, *P = 0.011). Data are presented as the mean ± s.e.m. Fifty neighboring cells each were analyzed for a total of 93 CD8^+^ T cells and 5,000 random cells. **j**, IHC of human hippocampal sections from 14 patients with AD showed CD8^+^ T cells (brown) in contact with MHC-II^+^ myeloid cells (blue). The box plot shows the percentage of CD8^+^ T cells in contact (<50 µm) with MHC-II^+^ cells in patients with AD (ABC score > 1). The central line represents the median, the boxes the upper and lower quartiles and the whiskers show the full range of the data. **e**, Scale bar, 10 µm. **j**, Scale bar, 50 µm. Source data

**Extended Data Fig. 6:**
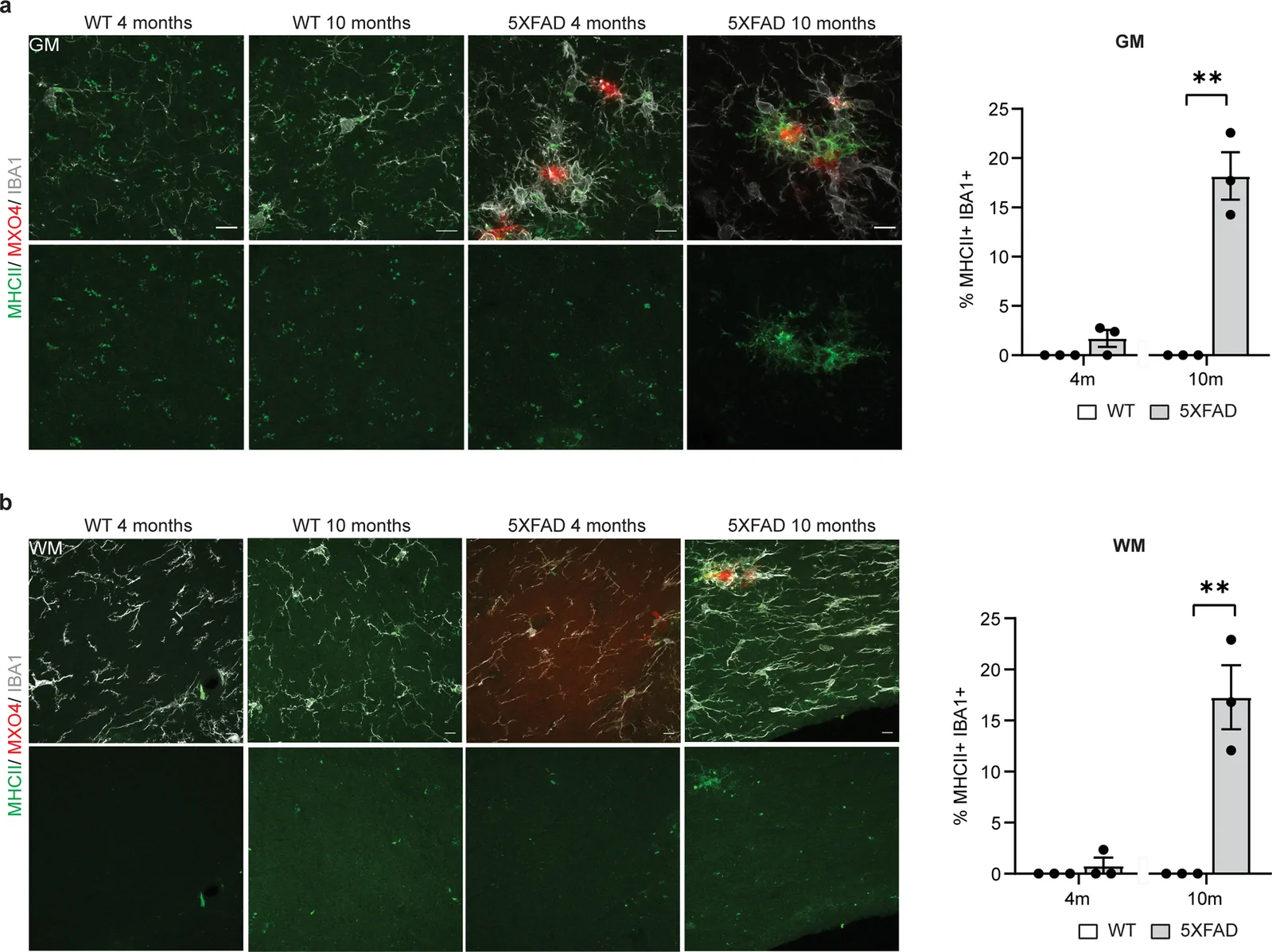
Characterization of MHCII^+^ cells in *5xFAD* mice. **a**, Representative image showing MHCII^+^ (green) IBA1^+^ cells (grey) in the cortex of WT and *5xFAD* mice aged 4 and 10 months. Plaques are stained with Methoxy-X04 (MX04, red). Scale bar, 10 µm. Quantifications indicate the percentage of IBA1^+^ cells also positive for MHCII. Statistical significance was determined for n = 3 animals using unpaired two-sided Student’s t-test (WT 10 m, *5xFAD* 10 m, **p = 0.001). **b**, Representative image showing MHCII^+^ (green) IBA1^+^ cells (grey) in the corpus callosum of WT and *5xFAD* mice aged 4 and 10 months. Plaques are stained with Methoxy-X04 (MX04, red). Scale bar, 10 µm. Quantifications indicate the percentage of IBA1^+^ cells also positive for MHCII. Statistical significance was determined for n = 3 animals using unpaired two-sided Student’s t-test (WT 10 m, *5xFAD* 10 m, **p = 0.005). Each point on the graph represents one animal. Data is presented as mean ± SEM. Source data

To understand the mechanisms of how CD8^+^ T cells may induce the injury, we evaluated its effect on microglia by performing single-cell RNA sequencing (scRNA-seq) using the 10X platform for 6-month-old 5xFAD mice treated with anti-CD8 or isotype control antibody. We obtained 40,394 single-cell transcriptomes (Supplementary Fig. 2). Cell type composition was analyzed using unsupervised uniform manifold approximation and projection (UMAP); cell type identities were confirmed by the expression of canonical cell type marker genes (Supplementary Fig. 2). Microglia were distributed in nine different clusters: a homeostatic cluster, IFN-responsive clusters and different disease-associated microglial (DAM) clusters as described previously^13–17^ (Fig. 2a,b). We used Monocle 3 for semisupervised pseudotime analysis to investigate whether the distinct responses followed a continuous differentiation pathway. Our findings revealed that microglia aligned themselves along a trajectory, transitioning from a homeostatic state over the IFN-responsive microglial (IRM) state toward the various DAM states (Supplementary Fig. 3a,c). Notably, the expression of major histocompatibility complex class II (MHC-II) genes was observed in the cells located at the furthest end of the trajectory. Slingshot analysis provided further evidence that the specific DAM subpopulation with high MHC-II gene represented an advanced activation stage (Supplementary Fig. 3d). Next, we investigated the impact of CD8^+^ T cell depletion on microglial responses. To achieve this, we used differential single-cell composition data analysis (scCODA) and found that the DAM cluster enriched in MHC-II genes was significantly decreased in CD8^+^ T cell-depleted 5xFAD mice (Fig. 2c,d). Using immunohistochemistry (IHC), we detected an increase in MHC-II^+^IBA1^+^ cells in 5xFAD mice with time (Extended Data Fig. 6), and consistent with the scRNA-seq data, we found that MHC-II^+^IBA1^+^ cells were significantly reduced by CD8^+^ T cell depletion in 5xFAD (Fig. 2e). Furthermore, *APP*^*NLGF*^ crossed with *Rag1* KO mice had fewer MHC-II^+^IBA1^+^ cells (Extended Data Fig. 5e). Checkpoint inhibition, in contrast, resulted in an increase in the number of MHC-II^+^IBA1^+^ cells in 5xFAD mice (Extended Data Fig. 4f). These results suggested that T cells coordinate the MHC-II and some of the IFN signature possibly by secreting cytokines and IFNs. To investigate the spatial organization of T cells and MHC-II and the IFN signature, we performed single-cell-resolved spatial transcriptomics analysis (Fig. 2f–i and Supplementary Fig. 4a,b). Specifically, we measured a panel of 496 genes in five half-brain sections from three different 5xFAD mice (9.5-month-old) using multiplexed error-robust fluorescence in situ hybridization (MERFISH) (Fig. 2f). We found that cells neighboring the T cells displayed higher levels of MHC-II genes and several IFN-stimulated genes (ISGs) compared to nonneighboring cells (Fig. 2h). We observed an elevated ISG or MHC-II score for cells neighboring T cells (Fig. 2g). Quantification revealed that approximately 18% of cells adjacent to T cells exhibited *Cd74* expression, in contrast to only 1% of neighboring cells around a random 4′,6-diamidino-2-phenylindol (DAPI)^+^ cell (Fig. 2i), which we confirmed using IHC (Supplementary Fig. 4c). Next, we used human autopsy samples and observed that the overall CD8^+^ T cell density in patient brain tissue (hippocampus) increased significantly with increasing neuropathological AD probability according to the National Institutes of Health criteria (ABC score) (Supplementary Fig. 4d). After this, we performed IHC for CD8 and MHC-II, and found that approximately 45% of CD8^+^ T cells were in close proximity (<50 µm) to MHC-II^+^ cells (Fig. 2j); the density of these CD8^+^ T and MHC-II^+^ cell pairs correlated with the number of amyloid plaques in the hippocampus (Supplementary Fig. 4e).

**Extended Data Fig. 7:**
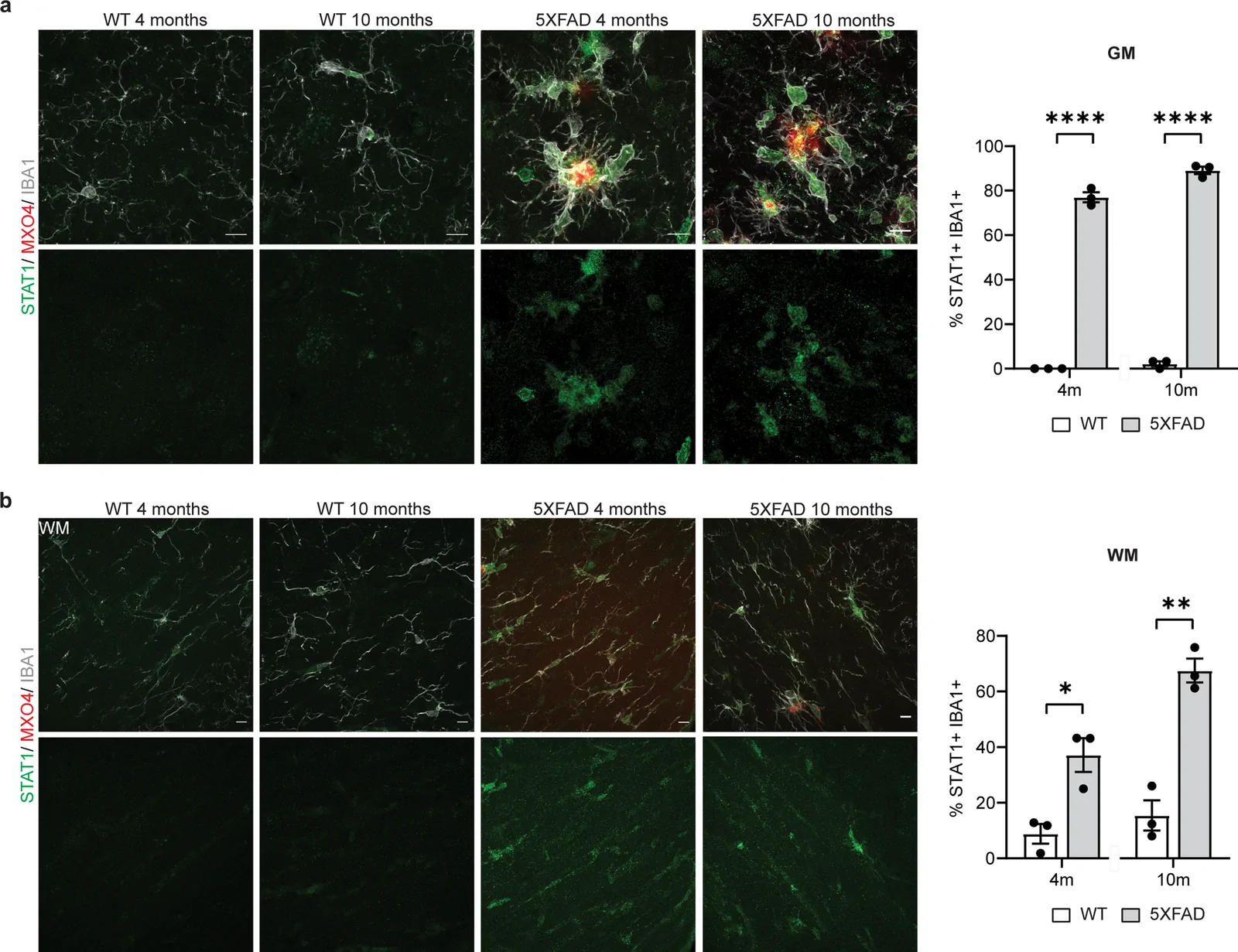
Characterization of STAT1^+^IBA1^+^ cells in *5xFAD* mice. **a**, Representative image showing STAT1^+^ (green) IBA1^+^ (grey) microglia in the cortex of WT and *5xFAD* mice aged 4 and 10 months. Plaques are stained with Methoxy-X04 (MX04, red). Scale bar 10 µm. Quantifications indicate the percentage of IBA1^+^ microglia also positive for STAT1. Statistical significance was determined for n = 3 animals using unpaired two-sided Student’s t-test (WT 4 m, *5xFAD* 4 m, ****p = 0.000004; WT 10 m, *5xFAD* 10 m, ****p = 0.000002). **b**, Representative image showing STAT1^+^IBA1^+^ cells in the corpus callosum of WT and *5xFAD* mice aged 4 and 10 months. Plaques are stained with Methoxy-X04 (MX04, red). Scale bar 10 µm. Quantifications indicate the percentage of IBA1^+^ microglia also positive for STAT1. Statistical significance was determined for n = 3 animals using unpaired two-sided Student’s t-test (WT 4 m, *5xFAD* 4 m, *p = 0.015; WT 10 m, *5xFAD* 10 m, **p = 0.001). Each point on the graph represents one animal. Data is presented as mean ± SEM. Source data

**Extended Data Fig. 8:**
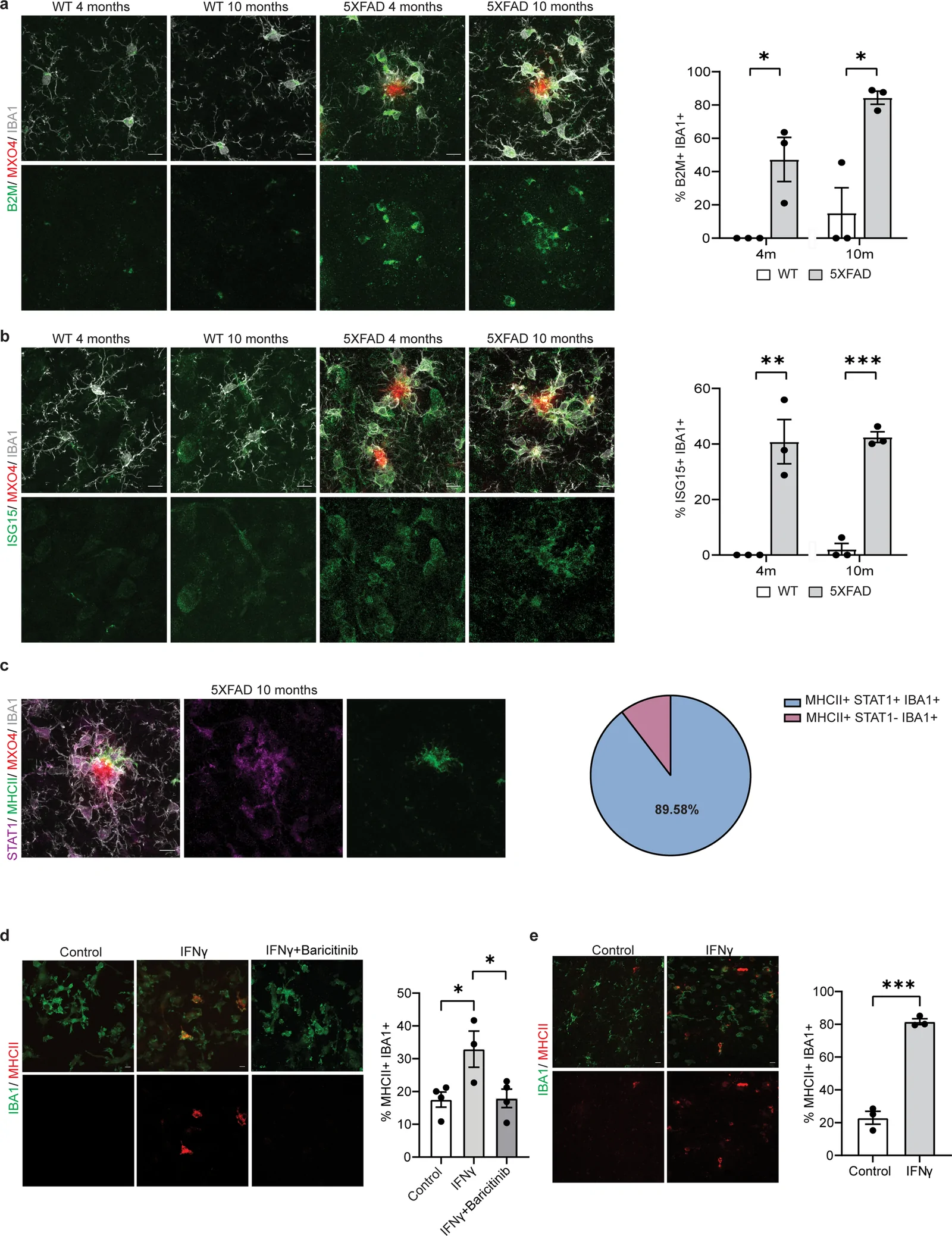
Characterization of IFNγ-activated microglia signatures in *5xFAD* mice. **a**, Representative image showing B2M^+^ (green) IBA1^+^ cells (grey) in the cortex of WT and *5xFAD* mice aged 4 and 10 months. Plaques are stained with Methoxy-X04 (MX04, red). Scale bar, 10 µm. Quantifications indicate the percentage of IBA1^+^ cells also positive for B2M. Statistical significance was determined for n = 3 animals using unpaired two-sided Student’s t-test (WT 4 m, *5xFAD* 4 m, *p = 0.023; WT 10 m, *5xFAD* 10 m, *p = 0.011). **b**, Representative image showing ISG15^+^ (green) IBA1^+^ cells (grey) in the cortex of WT and *5xFAD* mice aged 4 and 10 months. Plaques are stained with Methoxy-X04 (MX04, red). Scale bar, 10 µm. Quantifications indicate the percentage of IBA1^+^ cells also positive for ISG15. Statistical significance was determined for n = 3 animals using unpaired two-sided Student’s t-test (WT 4 m, *5xFAD* 4 m, **p = 0.006; WT 10 m, *5xFAD* 10 m, ***p = 0.0001). **c**, Representative image showing MHCII^+^ (green) STAT1^+^ (magenta) IBA1^+^ (green) cells. Plaques are stained with Methoxy-X04 (MX04, red). Scale bar, 10 µm. Pie chart shows mean percentage of MHCII^+^ IBA1^+^ cells positive or negative for STAT1 for n = 3 animals. **d**, Representative image showing primary microglia cultures treated with control (PBS), 1 ng/µL IFNγ and 1 ng/µL IFNγ with 1 µM baricitinib. Scale bar, 10 µm. Quantifications indicate the percentage of IBA1^+^ cells (green) also positive for MHCII (red). Statistical significance was determined for n = 3-4 biological replicates by one-way ANOVA with Tukey’s post hoc test (control, IFNγ, *p = 0.036; IFNγ, IFNγ+baricitinib, *p = 0.040). **e**, Representative image of old 24-month old mice injected with PBS (control) and 10 ng IFNγ. Scale bar, 10 µm. Quantifications indicate the percentage of IBA1^+^ cells (green) also positive for MHCII (red). Statistical significance was determined for n = 3 animals using unpaired two-sided Student’s t-test (control, IFNγ, ***p = 0.0002). Each point on the graph represents one animal. Data is presented as mean ± SEM. Source data

Because T cells are a major source of interferon-γ (IFNγ), and an effective inducer of MHC-II gene expression^18^, we asked whether MHC-II^+^IBA1^+^ cells were IFN-activated. Surprisingly, we detected that already at 4 months (when the plaque number is low compared to 10 months) a relatively large fraction of IBA1^+^ cells were positive for *STAT1*, *ISG15* and *B2M*, all of which represent IFN-responsive genes. This was even more prominent at 10 months of age (Extended Data Figs. 7 and 8a,b). Essentially, all MHC-II^+^IBA1^+^ cells were also STAT1^+^ (Extended Data Fig. 8c). To determine whether IFNγ could induce MHC-II expression, we treated cultured microglia with IFNγ, which resulted in the induction of MHC-II^+^ cells (Extended Data Fig. 8d). Likewise, injection of IFNγ into mice caused an increase in the formation of MHC-II^+^IBA1^+^ cells (Extended Data Fig. 8e). Together, our results suggest that CD8^+^ T cells amplify DAM responses by inducing the expression of a subset of cells characterized by high expression of MHC-II genes.

**Fig. 3:**
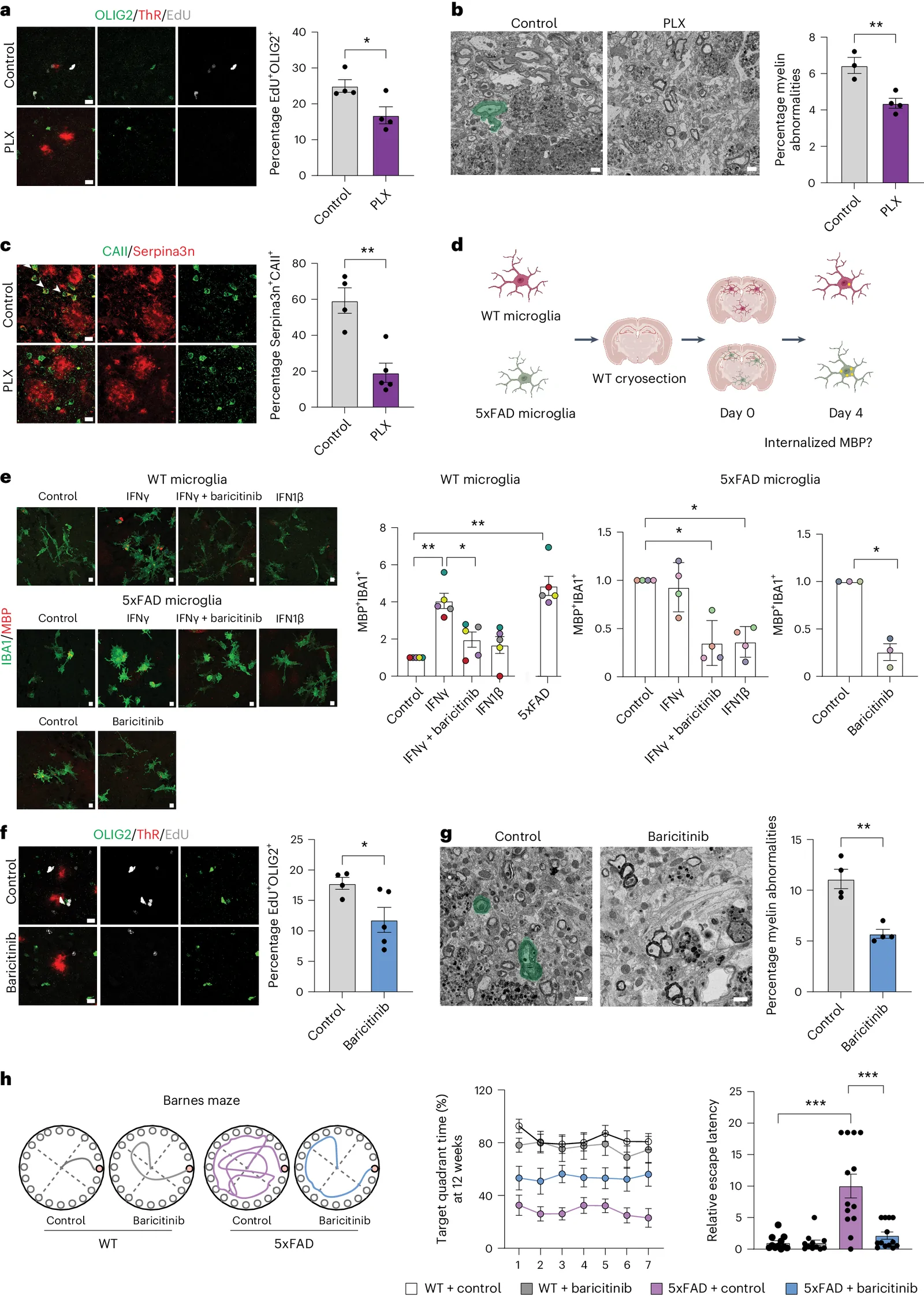
IFNγ-mediated microglial activation triggers myelin damage. **a**, Image and quantification of OLIG2^+^ (green) EdU^+^ (gray) cells in the cortex of control or PLX-treated mice. ThR (red) indicate plaques. Unpaired two-sided Student’s *t*-test for *n* = 4 mice (control, PLX, *P = 0.03). **b**, SEM images and quantification of myelin abnormalities (green) in the cortex of control or PLX mice. Unpaired two-sided Student’s *t*-test for *n* = 3–4 mice (control, PLX, **P = 0.008). **c**, Image and quantification of CAII^+^ (green) Serpina3n^+^ (red) oligodendrocytes in the cortex of control or PLX mice. Unpaired two-sided Student’s *t*-test for *n* = 4–5 mice (control, PLX, **P = 0.0024). **d**, Schematic of the assay. **e**, Image showing IBA1^+^ (green) cells with internalized MBP (red). Quantification indicated fold change in the number of MBP^+^IBA1^+^ cells normalized to control. Left and middle: One-way ANOVA with Tukey’s post hoc test. Right, Unpaired two-sided Student’s *t*-test for *n* = 3–5 independent experiments (WT microglia, control, IFNγ, **P = 0.008; WT microglia, IFNγ, IFNγ + baricitinib, *P = 0.016; WT microglia, 5xFAD microglia, **P = 0.008; 5xFAD microglia, control, IFNγ + baricitinib, *P = 0.034; 5xFAD microglia, control, IFN1β, *P = 0.012; 5xFAD microglia, control, baricitinib, *P = 0.013). **f**, Image and quantification of OLIG2^+^ (green) EdU^+^ (gray) cells in the cortex of 7.5-month-old control or baricitinib-treated mice. ThR (red) indicate plaques. Unpaired two-sided Student’s *t*-test for *n* = 4–5 mice (control, baricitinib, *P = 0.047). **g**, SEM images and quantification of myelin abnormalities (green) in the cortex of 9-month-old control or baricitinib-treated mice. Unpaired two-sided Student’s *t*-test for n = 4 mice (control, baricitinib, **P = 0.002). **h**, Behavior of 5xFAD mice in the Barnes Maze after 12 weeks of treatment with baricitinib. Left: Tracing graphs in the platform trials in the Barnes maze. Right: Quantification of time in the target quadrant (%) on the platform during training and relative escape latency to the target hole in the probe trial. One-way ANOVA with Tukey’s post hoc analysis for *n* = 11–13 mice (relative escape latency, 12 weeks, WT control, 5xFAD control, ***P = 0.00000431; relative escape latency, 12 weeks, 5xFAD control, 5xFAD baricitinib, ***P = 0.00002349). Data are presented as the mean ± s.e.m. **a**,**c**,**f**, Scale bar, 10 µm. **b**,**g**, Scale bar, 1 µm. Source data

To delineate the role of microglia in driving oligodendrocyte pathology, we performed pharmacological depletion of microglia by treating 6-month-old 5xFAD mice for 6 weeks with Plexxicon (PLX5622 (PLX)) or control chow. Notably, there was significant reduction in IBA1^+^ cells, EdU^+^OLIG2^+^ cells, Serpina3n^+^ oligodendrocytes and myelin alterations, whereas amyloid plaque numbers, CD8^+^ T cells and STAT1^+^CAII^+^ oligodendrocytes were unaltered (Fig. 3a–c and Supplementary Fig. 5). To further assess the effect of microglia activation on myelin in 5xFAD mice, we analyzed the number of microglia containing intracellular MBP fragments. We found an increase in the number of MBP^+^ microglia in 10-month-old 5xFAD mice, wherein most MHC-II^+^ microglia contained MBP fragments (Supplementary Fig. 6). Next, we asked whether such activated microglia in 5xFAD mice were more prone to induce myelin damage. To address this question, we established an ex vivo slice culture model in which brain cryosections from wild-type (WT) mice were seeded with microglia purified from 5xFAD or WT mice (Fig. 3d). Microglia from 5xFAD mice phagocytosed myelinated fibers, as detected by an increase in MBP^+^ material within microglia (Fig. 3e). This was in contrast to microglia purified from WT mice, which displayed hardly any internalized MBP^+^ material. However, when microglia from WT mice were incubated with IFNγ, we observed an increase in MBP^+^ material within microglia (Fig. 3e and Supplementary Fig. 7). IFNβ showed no observable effects. To determine whether the IFN pathway was responsible for the abnormal activation of microglia, we treated microglia with baricitinib, a Janus kinase (JAK) inhibitor that blocks JAK1 and JAK2 signaling. Baricitinib effectively reduced microglial activity toward myelin in the slice assay (Fig. 3e and Supplementary Fig. 7). Furthermore, baricitinib diminished IFNγ-mediated MHC-II induction in cultured microglia (Extended Data Fig. 8d). Collectively, these findings offer evidence that abnormal IFN activation may trigger microglia to engage in myelin-damaging activity.

**Extended Data Fig. 9:**
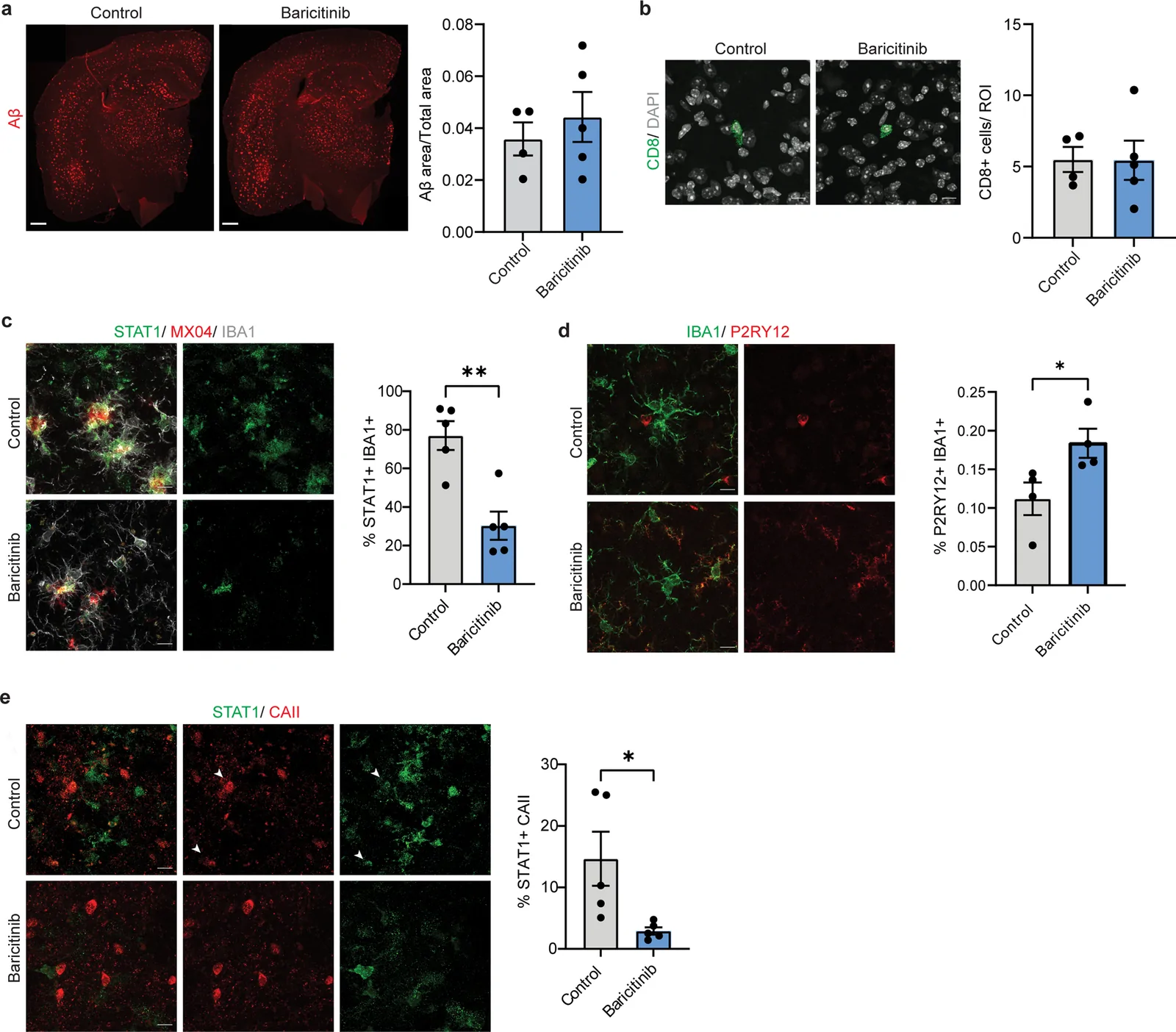
Baricitinib treatment results in no change in amyloid plaque load and CD8^+^ T cell numbers. **a**, Representative image showing Aβ plaque load (red) in 9 months old control and baricitinib treated mice. Quantifications indicate proportion of Aβ positive area normalised to total area in the cortex. Statistical significance was determined for n = 4-5 animals using unpaired Student’s t-test. **b**, Representative image showing CD8^+^ T cells (green) in the cortex of control and baricitinib treated mice (9 month-old). Scale bar, 10 µm. Quantifications indicate the number of CD8^+^ T cells in the cortex. Statistical significance was determined for n = 4-5 animals using unpaired Student’s t-test. **c**, Representative image showing STAT1^+^ (green) IBA1^+^ (grey) cells in control and baricitinib treated mice (7.5 months old). Plaques are stained with Methoxy-X04 (MX04, red). Scale bar, 10 µm. Quantifications indicate percentage of IBA1^+^ cells also positive for STAT1. Statistical significance was determined for n = 5 animals using unpaired two-sided Student’s t-test (control,baricitinib, **p = 0.002). **d**, Representative image showing P2RY12^+^ (red) IBA1^+^ (green) cells in the cortex of control and baricitinib treated mice (7.5 month-old). Scale bar, 10 µm. Quantifications indicate percentage of IBA1^+^ cells also positive for P2RY12. Statistical significance was determined for n = 4 animals using unpaired two-sided Student’s t-test (Control, Baricitinib, *p = 0.04). **e**, Representative image showing STAT1^+^ (green) CAII^+^ (red) oligodendrocytes in the cortex of control and baricitinib treated mice (7.5 month-old). Scale bar, 10 µm. Quantifications indicate percentage of CAII^+^ cells also positive for STAT1. Statistical significance was determined for n = 5 animals using unpaired two-sided Student’s t-test (control, baricitinib, *p = 0.0296). Each point on the graph represents one animal. Data is presented as mean ± SEM. Source data

**Extended Data Fig. 10:**
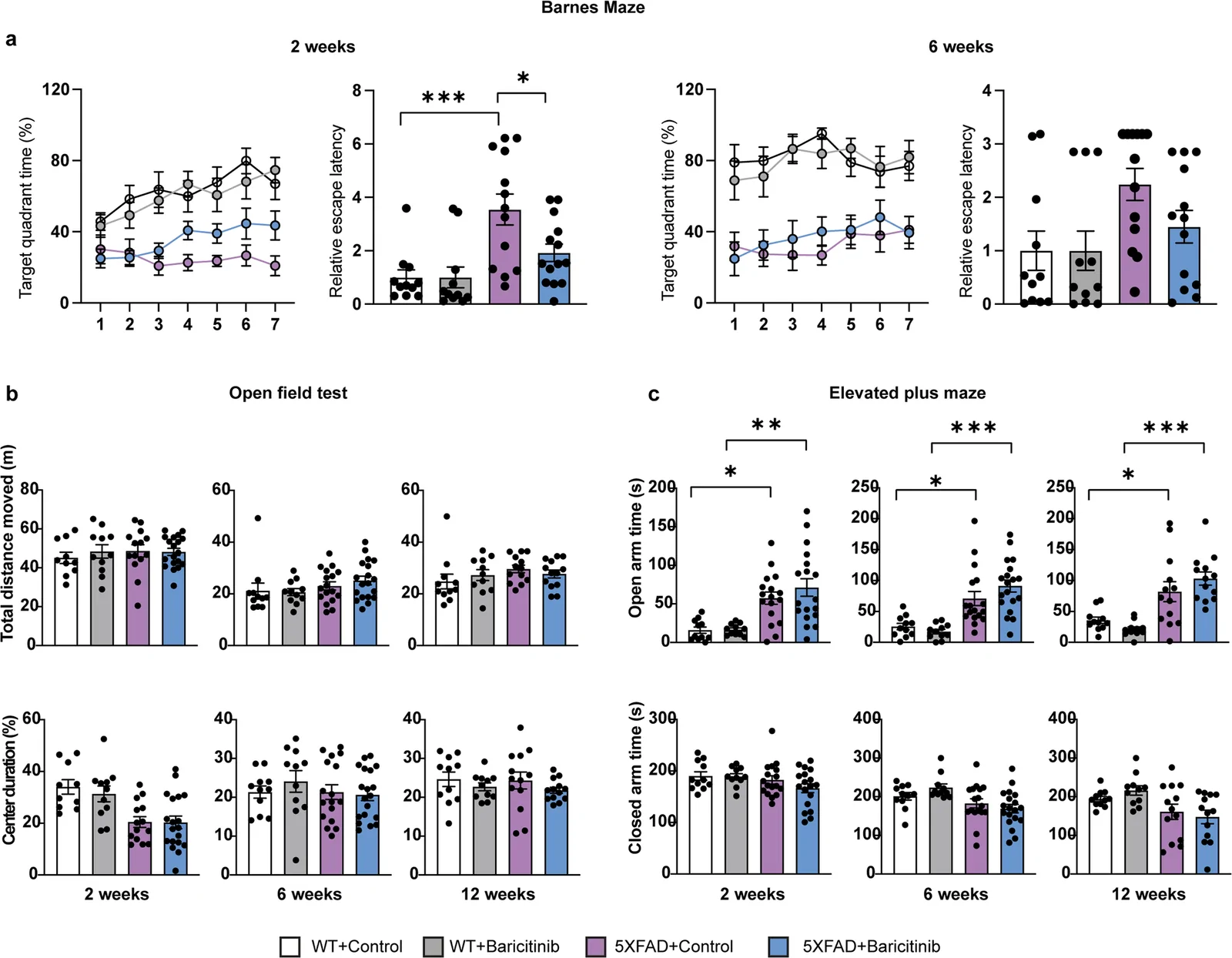
Effects of baricitinib treatment on behavior in *5xFAD* mice. Behavior of *5xFAD* mice in Barnes Maze following 2 weeks and 6 weeks of treatment with baricitinib. Quantification of the target quadrant time (%) on the platform during the training sessions and relative escape latency to the target hole in probe trial. Statistical significance was evaluated for n = 11–14 animals by one-way ANOVA followed by Tukey’s post hoc analysis using paired t-test (Relative escape latency, 2 weeks, WT control, *5xFAD* control, ***p = 0.0007; Relative escape latency, 2 weeks, *5xFAD* control, *5xFAD* baricitinib, *p = 0.0321). **b**, Behavior of *5xFAD* mice in an Open Field test following 2 weeks, 6 weeks and 12 weeks of treatment with baricitinib. Quantification of distance travelled on the platform (upper panel) and the percentage of time spent in center (lower panel). Statistical significance was evaluated for n = 10–19 animals by one-way ANOVA followed by Tukey’s post hoc analysis using paired t-test. **c**, Behavior of *5xFAD* mice in an Elevated Plus Maze test following 2 weeks, 6 weeks and 12 weeks of treatment with baricitinib. Quantification of time spent in open arms (upper panel) and the time spent in close arms (lower panel). Statistical significance was evaluated for n = 11–19 animals by one-way ANOVA followed by Tukey’s post hoc analysis (2 weeks, WT control, *5xFAD* control, *p = 0.0115; 2 weeks, WT baricitinib, *5xFAD* baricitinib, **0.0004; 6 weeks, WT Control, *5xFAD* Control, *p = 0.0125; 6 weeks, WT baricitinib, *5xFAD* baricitinib, ***p = 0.00001283; 12 weeks, WT Control, *5xFAD* Control, *p = 0.02; 12 weeks, WT baricitinib, *5xFAD* baricitinib, ***p = 0.00002062). Each point on the graph represents one animal. Data is presented as mean ± SEM. Source data

Thus, we tested the ability of baricitinib to reduce myelin damage in 5xFAD mice in vivo. Six-month-old 5xFAD mice were treated for up to 12 weeks with baricitinib (10 mg kg^−1^ body weight). We observed a reduction in STAT1^+^CAII^+^ oligodendrocytes and STAT1^+^IBA1^+^ microglia, with a concomitant increase in P2RY12^+^IBA1^+^ cells in 5xFAD mice. No significant differences in amyloid plaque load and CD8^+^ T cell numbers were observed (Extended Data Fig. 9a,b). However, there was a decrease in the number of EdU^+^OLIG2^+^ cells (Fig. 3f), and by using EM, fewer myelin abnormalities were detected in baricitinib-treated 5xFAD mice (Fig. 3g). To assess learning deficits and anxiety-related behaviors, we performed the Barnes maze, elevated plus maze and open field tests. Baricitinib treatment resulted in a significant improvement of learning deficits in 5xFAD mice, whereas anxiety-related behaviors were unchanged (Fig. 3h and Extended Data Fig. 10).

Collectively, our data provide evidence for the progressive accumulation of abnormally activated microglia in 5xFAD mice that display myelin-damaging activity. The activity of infiltrating CD8^+^ T cells previously detected in models of AD^19,20^ could be responsible for driving microglia into such a state. Progressive myelin damage has the properties of a self-propelling positive feedback system in which more myelin pathology causes greater inflammation and the next loop causes an even greater response. If these results are validated further in human studies, targeting inflammation for the promotion of oligodendrocyte health could hold significant therapeutic promise for delaying AD.

## Methods

### Mice

All animal experiments were performed with the approval and according to the regulations of the District Government of Upper Bavaria and reported according to the guidelines. Animals were housed at the German Center for Neurodegenerative Diseases (DZNE) in Munich in groups in Greenline IVC GM500 plastic cages in a temperature-controlled environment (21 ± 2 °C) on a 12-h light–dark cycle with food and water available ad libitum. The mouse lines used in the study include: WT C57BL/6J mice from Janvier Labs, B6.Cg-Tg(APPSwFlLon,PSEN1*M146L*L286V)6799Vas/Mmjax (5xFAD)^21^ and B6.129S7-*Rag1*^*tm1Mom*^/J (*Rag1* KO)^22^ from the Jackson Laboratory, and App^tm3.1Tcs^ (*APP*^*NLGF*^)^23^. Experiments were performed with adult male mice aged 4, 6, 10 and 12 months as indicated in the figures and figure legends. Mice were randomly assigned to different treatment groups. For the depletion of CD8^+^ T cells, male mice, 5xFAD mice aged 6 months were injected intraperitoneally with anti-CD8 antibody (catalog no. BP0061, Bio X Cell) and their respective isotype control (catalog no. BE0119, Bio X Cell) twice a week for a total of 6 weeks. 5xFAD male mice aged 6 months were given either antibodies against PD-1 (catalog no. BP0146, Bio X Cell) and CTLA4 (catalog no. BP0131, Bio X Cell) or their isotype controls (catalog nos. BE0089 and BP0087, Bio X Cell) at a concentration of 10 mg kg^−1^ or 20 mg kg^−1^ body weight, respectively. Antibodies were injected intraperitoneally twice a week for 6 weeks. WT and 5xFAD female mice aged 6 months were fed powdered chow (ssniff V1120) mixed with baricitinib (catalog no. V0338, InvivoChem) dissolved in corn oil at a concentration of 10 mg kg^−1^ body weight. Alternatively, control animals were fed with powdered chow mixed with corn oil only. 5xFAD female mice aged 6 months were fed either with control AIN-76A standard chow or 0.12% PLX5622 (MedChemExpress, mixed in with AIN-76A standard chow, Research Diets) for 6 weeks. EdU (catalog no. E10187, Invitrogen) was dissolved in drinking water at a final concentration of 0.2 mg ml^−1^ for 2 weeks. Water was replaced every 48 h.

### Human samples

Patients with a neuropathological diagnosis of AD were retrieved from the archives of the Institute of Neuropathology, University Medical Center Göttingen, Germany. Patients were eligible to participate in the study if they or their next of kin had consented to tissue donation and to the use of their tissue for research. The study was approved by the ethics committee of the University Medical Center Göttingen (39/2/19). Sampling of the hippocampus was performed during autopsy in 16 individuals; samples were formalin-fixed and paraffin-embedded. Each individual was stratified according to the ‘ABC’ score^24^. Patients with intermediate or high risk (*n* = 12) were assigned as patients with AD. For patient information, see Supplementary Table 1.

### IHC

Mice were anesthetized with a solution of 10 mg ml^−1^ ketamine and 1 mg ml^−1^ xylazine injected intraperitoneally. They were then perfused transcardially with 4% paraformaldehyde (PFA) and brains were post-fixed in 4% PFA for 2 h, followed by cryopreservation in 30% sucrose in PBS. After freezing the tissue on dry ice using Tissue-Tek O.C.T. compound, 30-μm coronal sections were cut using the Leica CM 1900 cryostat. Free-floating tissue sections were collected in a solution containing 25% glycerol and 25% ethylene glycol in PBS. Tissue sections were rinsed with 1× PBS followed by permeabilization with 0.5% Triton X-100 in PBS for 30 min at room temperature. The sections were then incubated in Fab fragment goat anti-mouse IgG (1:100 dilution, Dianova) for 1 h at room temperature to block endogenous mouse tissue immunoglobulins. Sections were blocked for 1 h in a solution consisting of 2.5% FCS, 2.5% fish gelatin and 2.5 % fish gelatin in PBS. Primary antibodies were diluted in 10% blocking solution and incubated overnight at 4 °C. The following day, sections were washed in PBS and then incubated in secondary antibodies in 10% blocking solution for 2 h at room temperature. The sections were then washed with 1× PBS and stained with DAPI for 5 min and mounted. The following antibodies were used: mouse anti-APC (1:100 dilution, catalog no. OP80-100UG, Merck Millipore), rabbit anti-B2m (1:100 dilution, ab75853, Abcam), rabbit anti-STAT1 (1:100 dilution, catalog no. 14994S, Cell Signaling Technology), rat anti-CD8 (1:100 dilution, catalog no. 100702, Promega Corporation), rabbit anti-Iba1 (1:250 dilution, catalog no. 234-004, Wako), chicken anti-IBA1 (1:400 dilution, catalog no. 234009, Synaptic Systems), mouse anti-β-Amyloid1-11 (1:1,000 dilution, catalog no. 835104, BioLegend), mouse anti-myelin-associated glycoprotein (1:200 dilution, catalog no. MAB1567, Sigma-Aldrich), mouse anti-Serpin A3N (1:100 dilution, catalog no. AF4709, Bio-Techne), rabbit anti-Olig2 (1:250 dilution, catalog no. AB9610, Sigma-Aldrich), mouse anti-PDGF R alpha (1:100 dilution, catalog no. AF1062, R&D Systems), rat anti-MHC Class II (1:100 dilution, catalog no. 14-5321-82, Invitrogen), mouse anti-ISG15 (1:100 dilution, catalog no. sc-166755, Santa Cruz Biotechnology), rat anti-myelin basic protein (1:300 dilution, catalog no. ab7349, Abcam), chicken anti-MBP (1:400 dilution, catalog no. PA1-10008, Invitrogen), rabbit anti-BCAS1 (1:500 dilution, generated against amino acids 1–667 of mouse BCAS1, custom-made by Synaptic Systems), donkey anti-mouse IgG (H+L) Alexa Fluor 488 (1:500 dilution, catalog no. A-21202, Thermo Fisher Scientific), goat anti-mouse IgG (H+L) Alexa Fluor 555 (catalog no. 1:500, catalog no. A-21422, Thermo Fisher Scientific), goat anti-mouse IgG (H+L) Alexa Fluor 647 (1:500 dilution, catalog no. A-21235, Thermo Fisher Scientific), goat anti-rabbit IgG (H+L) Alexa Fluor 488 (1:500 dilution, catalog no. A-11008, Thermo Fisher Scientific), goat anti-rabbit IgG (H+L) Alexa Fluor 555 (1:500 dilution, catalog no. A-21428, Thermo Fisher Scientific), goat anti-rat IgG (H+L) Alexa Fluor 555 (1:500 dilution, catalog no. A-21434, Thermo Fisher Scientific), goat anti-mouse IgG Alexa Fluor 555 (1:500 dilution, catalog no. A-32116, Thermo Fisher Scientific), donkey anti-goat IgG (H+L) Alexa Fluor Plus 647 (1:500 dilution, catalog no. A32849, Thermo Fisher Scientific), donkey anti-rat Alexa Fluor 488 (1:500 dilution, Thermo Fisher Scientific), donkey anti-goat Alexa Fluor 555 (1:500 dilution, Thermo Fisher Scientific) and donkey anti-rabbit Alexa Fluor 647 (1:500 dilution, Thermo Fisher Scientific). For CC1, STAT1 and Serpina3n antibodies, after permeabilization tissue sections were boiled in 10 mM citrate buffer, pH 6.0, at 85 °C for 20 min. To detect plaques, after incubation with secondary antibodies, sections were stained using either ThR or Methoxy-XO4. For ThR (catalog no. S570435, Sigma-Aldrich) sections were incubated at a concentration of 1:1,000 in 1× PBS for 15 min at room temperature. For Methoxy-XO4 (catalog no. 4920, Tocris) staining, sections were incubated with 4 µg ml^−1^ Methoxy-XO4 in 50% ethanol and washed with 50% ethanol for 5 min. To detect EdU, the Alexa Fluor 647 Click-iT EdU Cell Proliferation Assay Kit (catalog no. C10340, Invitrogen) was used according to the manufacturer’s protocol. Briefly, after incubation in secondary antibodies, brain sections were washed with PBS and incubated in EdU developing cocktail for 45 min at room temperature, followed by post staining with DAPI before mounting on coverslips. Images were acquired using a Leica Microsystems TCS SP5 confocal microscope or with an LSM 900 microscope (ZEISS) and were processed and analyzed with the Imaris v.9.2.0 and ImageJ v.1.41 software. For IHC of human samples, we performed heat antigen retrieval after deparaffinization (steamer, citrate buffer, pH 6, for 30 min), incubation in 3% H_2_O_2_ and blocking with normal goat serum (10%) with 0.1% Triton X-100. The primary mouse anti-CD8 antibody (1:50 dilution, clone C8/144B, catalog no. M7103, Dako) was incubated overnight and developed with 3,3′-diaminobenzidine (Dako REAL EnVision, catalog no. K500711-2, Dako) until saturation of the chromogenic reaction. The second primary mouse anti-MHC-II antibody (1:50 dilution, clone CR3/43, catalog no. M0775, Dako) was again incubated overnight and developed using a goat anti-mouse IgG (1:50 dilution, catalog no. 115-055-003, Jackson ImmunoResearch) with alkaline phosphatase and fast blue as chromogen. Stained samples were digitized using a VS200 slide scanner (Evident) and manually quantified using the OMERO.iviewer software. CD8 cells were considered in the contact zone of an MHC-II^+^ cell if within a range of 50 µm.

### Cell culture

Microglia were isolated from P15-P21 C57BL/6J WT pups using the Neuronal Tissue Dissociation Kit (Miltenyi Biotec) according to the manufacturer’s protocol. Briefly, brain tissue was dissected, cut into small pieces and dissociated using enzymatic digestion in the gentleMACS Octo Dissociator (Miltenyi Biotec). The tissue suspension was incubated with magnetic beads against CD11b and passed through a magnetic column. Microglia were flushed out of the column and plated in DMEM containing 10% FCS, 10 ng ml^−1^ monocytic colony-stimulating factor, 1% penicillin-streptomycin and 1% glutamate for 4–7 days before being used for the experiments.

### Ex vivo myelin phagocytosis assay

Acute isolation of adult WT and 5xFAD microglia was performed as described above. In preparation for the assay, WT brains were flushed with PBS and fresh-frozen. Frozen brains were cut into 10-µm sections and transferred on Poly-l-lysine-coated coverslips and stored at −80 °C until use as described previously^25^. Frozen sections were temperature-adjusted for 30 min, washed with PBS and equilibrated to the microglial medium. Then, 100,000 acutely isolated microglia were plated on sections and immediately treated with 5 ng ml^−1^ IFNγ, 1 µM baricitinib or 5,000 U ml^−1^ IFN1β. Microglia were incubated on brain sections for 4 days; then, the internalized MBP^+^ material was analyzed.

### Stereotactic injection in the corpus callosum

A solution of 10 ng μl^−1^ IFNγ was prepared in sterile 1× PBS. To identify the lesion area during tissue processing, Monastral blue (autoclaved and filtered, catalog no. 274011, Sigma-Aldrich) was added to a final concentration of 0.03%. Mice were anesthetized using a solution of medetomidine (concentration 0.5 mg kg^−1^), midazolam (concentration 5 mg kg^−1^) and fentanyl (concentration 0.05 mg kg^−1^) injected intraperitoneally. After anesthesia, the head was shaved and bepanthen cream (catalog no. 1578847, Bayer) was used to protect the eyes. The mouse was positioned on the stereotactic injection apparatus and a small incision was made in the skin to expose the skull. A small hole was drilled into the skull at the injection coordinates: *X*, ±0.55 mm; *Y*, −1.22 mm (from bregma); Z, −1.25 mm (from bregma). A glass capillary containing an IFNγ solution was lowered to the desired coordinates and 1 µl IFNy was injected at a speed of 100 nl min^−1^. After the injection, the mouse was injected with buprenorphine (concentration 0.05 mg per kg of body weight) and the skin was sutured. To terminate the anesthesia, the mouse was injected with an antagonist solution consisting of atipamezole (concentration 2.5 mg kg^−1^), naloxone (concentration 0.5 mg kg^−1^) and flumazenil (concentration 0.5 mg kg^−1^). The animals were perfused transcardially after 48 h with 4% PFA as described above.

### EM

For the ultrastructural analysis, mice were perfused with 4% PFA (EM grade, Science Services) and 2 mM calcium chloride in 1× PBS, pH 7.4 (Science Services). One hemisphere was dedicated to ultrastructural analysis using immersion fixation in 4% PFA, 2.5% glutaraldehyde (EM grade, Science Services) and 2 mM calcium chloride in 0.1 M sodium cacodylate buffer for 24 h. Coronal 50–100-µm-thick vibratome sections were incubated in the same fixative for another 24 h and stored in 0.1 M sodium cacodylate buffer at 4 °C. The rOTO en bloc staining protocol included postfixation in 1% osmium tetroxide (Electron Microscopy Sciences), 1.5% potassium ferricyanide (Sigma-Aldrich) in 0.1 M sodium cacodylate (Science Services) buffer (pH 7.4)^26^. The contrast was enhanced by incubation in 1% thiocarbohydrazide (Sigma-Aldrich) for 45 min at 40 °C. The tissue was washed in water and incubated in 1% aqueous osmium tetroxide, washed and further contrasted using overnight incubation in 1% aqueous uranyl acetate at 4 °C and 2 h at 50 °C. Samples were dehydrated in an ascending ethanol series and infiltrated with the Araldite epoxy resin LX112 (Ladd Research). Blocks were cured for 48 h, trimmed (TRIM2, Leica Microsystems) and sectioned at 100-nm thickness using a 35° Ultra Diamond Knife (DiATOME) on an ultramicrotome (UC7, Leica Microsystems). Sections were collected onto 1× 0.5 cm carbon nanotube tape strips (Science Services) for SEM analysis. For SEM imaging, the samples on tape were attached to adhesive carbon tape (Science Services) on 4-inch silicon wafers (Siegert Wafer) and grounded using adhesive carbon tape strips (Science Services). EM micrographs were acquired on a Crossbeam Gemini 340 SEM (ZEISS) with a four-quadrant backscatter detector at 8 kV using ATLAS 5 Array Tomography (Fibics). Lateral medium-resolution images (100 nm) allowed the identification of regions of interest that were in turn reimaged at 4–10-nm lateral resolution. Image analysis was performed in Fiji^27^.

### Mice perfusion and cell isolation for the 10X Genomics experiments

For the 10X Genomics experiments, mice were deeply anesthetized and perfused with cold PBS. Each brain was removed and individually microdissected under a dissection microscope; the cortex and hippocampus were isolated from the brain and the attached choroid plexus was removed. Cells were isolated with a previously established isolation protocol^28^ using gentleMACS with the Papain Neural Tissue Dissociation Kit (Miltenyi Biotec) and actinomycin D (catalog no. A1410, Sigma-Aldrich) at a final concentration of 45 mM. After dissociation, myelin debris was removed from the cell suspension using Myelin Removal Beads II (Miltenyi Biotec). Cells were resuspended in 0.04% BSA + PBS and counted using an automated cell counter (TC20, Bio-Rad Laboratories) before loading onto the Chromium Controller.

### Library preparation for the 10X Genomics experiments

Single-cell suspensions were loaded on to the Chromium Single Cell Controller using the Chromium Single Cell 3′ Library & Gel Bead Kit v.3.1 (10X Genomics) chemistry according to the manufacturer’s instructions. Sample processing and library preparation were performed according to the manufacturer’s instructions using AMPure beads (Beckman Coulter). Libraries were sequenced on the DNBSEQ sequencing system (Dresden).

### Preprocessing and analyses of the 10X Genomics data

The raw sequencing data of each library were processed using Cell Ranger (10X Genomics) v.3.0 to generate the gene-by-cell unique molecular identifier (UMI) count matrix. The single-cell gene expression matrix was imported into the R package Seurat (v.4.3.0). For further analysis, cells were excluded according to the total UMI count (<500 or ≥50,000); the total detected gene number (≥7,500); and the mitochondrial UMI percentage (≥10%). To prevent the influence of the technical characteristics of the downstream analyses, we performed SCTransform to normalize the clean UMI matrix. Single-cell clustering was done according to the vignette (https://github.com/satijalab/seurat/blob/HEAD/vignettes/sctransform_vignette.Rmd). Variable features with a residual variance of more than 1.4 were selected; principal component analysis (PCA) was performed to normalize the matrix. Cluster markers were identified using the FindAllMarkers function. Microglia subclusters were identified using the same parameters described above. Microglia subcluster compositional analysis was done using scCODA v.0.1.9 according to the online vignette (https://sccoda.readthedocs.io/en/latest/getting_started.html). To capture nuanced yet biologically significant alterations, the false discovery rate was set at 0.4, as described in the vignette.

### Single-cell trajectory construction

The clean UMI matrix of all microglia was imported into the R package Monocle3 and processed using normalization and PCA analysis. The functions learn_graph and order_cells were used to construct the trajectory. Homeostatic microglia were set as the start point of the trajectory. The function choose_graph_segments was used to determine the path, with homeostatic microglia as the start point and DAM4 as the end point. The Moran’s I test was performed in the function graph_test to find differentially expressed genes (*q* < 0.05 and Moran’s index > 0.3) across the selected path. For the slingshot trajectory analysis, the clean UMI matrix of all microglia was imported into the R package SingleCellExperiment and processed using normalization and PCA analysis. A function slingshot was used to construct the trajectory without an indicated start or end point.

### MERFISH

#### Tissue collection and preparation for MERFISH

Male 5xFAD and WT litter-mate mice aged 9.5 months were anesthetized using an isoflurane chamber and film container filled with isoflurane-soaked cotton fibers and perfused with RNase-free PBS (catalog no. D8537, Sigma-Aldrich). Next, brains were carefully extracted, cut in half and frozen in isopentane on dry ice. Brains were then stored at −80 °C until further processing.

#### Tissue sectioning

Brain sections, 10 μm in thickness, were cut coronally using a cryotome (CryoStar NX70, Thermo Fisher Scientific) and collected onto glass slides supplied by Vizgen. To ensure proper adhesion, the glass slides were coated with a solution of 0.1 mg ml^−1^ poly-d-lysine bromide (catalog no. P7886, Sigma-Aldrich), left to incubate at room temperature for 1 h and dried before starting the sectioning.

#### Gene panel

The gene panel used in this study included 496 protein-coding genes, complemented by 54 blank probes. Within this gene panel, a diverse array of genes was selected, including recognized markers associated with brain and immune cell types, such as glial cells, vascular cells, T cells and macrophages, as well as distinct subtypes of glutamatergic and GABAergic neurons. Moreover, an assortment of reactive markers for microglia, astrocytes and oligodendrocytes were included, drawing from the existing literature; genes pertaining to cholesterol metabolic pathways and AD-related pathways were incorporated into the panel. For a comprehensive listing of the entire gene panel, see Supplementary Table 2.

#### Hybridization

After sectioning, samples were mounted on slides and transferred to Petri dishes (60 × 15 mm), with the tissue side facing upwards. These dishes were stored at the rear of the cryostat, maintaining a temperature of −20 °C for a minimum duration of 5 min to ensure adherence. Next, 5 ml fixation buffer (4% PFA in 1× RNase-free PBS) was gently added to the Petri dishes within a fume hood, followed by a 15-min incubation period at room temperature. Afterwards, samples were washed with 5 ml RNase-free PBS, three times each for 5 min. Samples were then permeabilized in 5 ml 70% ethanol at 4 °C overnight, in parafilm-sealed dishes, and kept under the same conditions for long-term storage (up to 1 month). For hybridization with the library (‘gene panel’), samples were washed with 5 ml Vizgen Sample Prep Wash Buffer and then incubated in 5 ml Formamide Hybridization Buffer at 37 °C for 30 min in an incubator. The Formamide Hybridization Buffer was aspirated from the tissue and 50 μl of the gene panel mix was added on top of each tissue. A piece of parafilm with an approximate size of 1.5 × 1.5 cm was carefully added on top of the liquid to spread the library mix and protect it from evaporation. The dishes were sealed with parafilm and placed in a humidified incubator at 37 °C for 36–48 h. The parafilm was removed and the samples were incubated twice in 5 ml Formamide Hybridization Buffer at 47 °C for 30 min. The samples were then washed with 5 ml Sample Prep Wash Buffer for 2 min.

#### Gel embedding

A fresh 10% w/v ammonium persulfate solution was prepared. For each sample, 5 ml of Gel Embedding Premix was combined with 25 μl of the 10% ammonium persulfate solution and 2.5 μl of N,N,N′,N′-tetramethylethylenediamine. In parallel, one 20-mm gel-coated coverslip for each sample was cleaned with RNaseZap, followed by 70% ethanol and dried with Kimwipes. The gel-coated coverslips were then covered with 100 μl Gel Slick Solution (VWR) for 10 min and wiped dry with Kimwipes. The Sample Prep Wash Buffer was aspirated from the samples. For each sample, 100 μl of the Gel Embedding Mix was retained in a small tube, while the remainder was added to the samples and incubated for 1 min. The Gel Embedding Mix was then poured out from the samples into a waste tube but kept aside on the bench (to monitor gel formation). The slides were then aspirated dry, leaving just enough liquid to keep the tissue from drying out; 50 μl of the retained Gel Embedding Mix was added on top of the tissue and the Gel Slick-treated coverslip was placed on top of it using tweezers, with the Gel Slick-treated side facing down toward the tissue, avoiding air bubbles. Excess Gel Embedding Solution was aspirated from the sides of the coverslips. The samples were then incubated at room temperature for 1.5 h to allow the gels to form. Finally, the coverslips were removed using a hobby blade and tweezers.

#### Tissue clearing

For each sample, to eliminate lipids and proteins that would interfere with imaging, 5 ml of the Clearing Premix were mixed with 50 μl Proteinase K. After the coverslips were removed from the gel-embedded samples, the clearing solution was added to each sample and the dishes were sealed with parafilm. Samples were placed in a humidified incubator overnight at 37 °C. Samples were stored in the Clearing Premix solution in the 37 °C incubator before imaging for up to a week. Samples were imaged after tissue could no longer be observed in the gel.

#### Sample imaging

The Clearing Premix solution was aspirated from the sample and the sample was briefly washed three times with Sample Prep Wash Buffer, then again for 10 min on a rocker and then three more times briefly. The sample was incubated with 3 ml of the appropriate first hybridization buffer, including DAPI and polyT reagent, for 15 min at room temperature on a rocker, covered from light. The sample was then washed with 5 ml of the Formamide Hybridization Buffer for 10 min at room temperature on a rocker, covered from light and then transferred to 5 ml of the Sample Prep Wash Buffer. The imaging buffer was prepared by combining the iImaging buffer, the imaging buffer activator and the RNase inhibitor at a ratio of 500:2.5:1. The hybridization buffers appropriate to the gene panel, and the imaging buffers, were loaded onto the MERSCOPE system (Vizgen). The sample was placed in the flow chamber and connected to the fluidics system of the MERSCOPE system (Vizgen), taking care to get rid of air bubbles. A low-resolution mosaic was acquired using a ×10 objective; regions of interest (whole coronal sections) were selected for high-resolution imaging with a ×60 lens. For high-resolution imaging, the focus was locked to the fiducial fluorescent beads on the coverslip. Seven 1.5-μm-thick Z-planes were taken for each field of view when imaging the tissue, including for the DAPI channel.

### MERFISH data analysis

Raw images were decoded to RNA spots with spatial coordinates and gene IDs using the Merlin software (Vizgen) on the MERSCOPE instrument. Cell segmentation was performed using CellPose algorithm, using the DAPI nuclear and polyT total RNA staining channels. The resulting single-cell gene expression matrices were further analyzed in R. We performed quality control for each section and excluded cells with fewer than 30 transcripts or fewer than ten detected genes. Data were then normalized using SCTransform v.2 normalization; PCA was calculated based on all 496 measured genes. Next, the dataset was harmonized for batch effect differences between sections using Harmony (with 75 principal components as the input). UMAP embedding was computed from the first 40 Harmony dimensions using the RunUMAP function. We calculated the shared nearest neighbor graph based on the first 40 Harmony dimensions (FindNeighbors function), which was then used to detect clusters using a Louvain algorithm at a range of resolutions. We annotated cells into major classes based on the coarse clustering resolution (0.1), and spatial location and regional identity. To identify T cells, we further subclustered the immune cell clusters by repeating the PCA, and using graph construction and clustering steps, to reveal a cluster marked by T cell marker genes (*Ptprc*, *Lck*, *Cd3e*, *Cd8a*, *Nkg7*), which we added to the cell type annotation. We used the AddModuleScore function to calculate the ISG expression score from the set of ten known ISGs included in our panel (*Stat1*, *Isg15*, *Rsad2*, *Usp18*, *Ifit1*, *Irf7*, *Ifitm3*, *Gbp2*, *Bst2*, *Cxcl10*). In the same manner, we calculated the MHC-II expression score based on Cd74, H2-Aa and H2-Ab1. To identify differentially expressed genes in the T cell neighborhoods, we calculated 50 nearest neighbors of every T cell based on two-dimensional spatial coordinates of cell centroids. We then identified differentially expressed genes between T cell neighbors and the remaining cells (nonneighbors) using the FindMarkers function with a Wilcoxon rank-sum test.

### Behavioral tests

All behavioral experiments were done and analyzed by experimenters who were blinded to the treatments and the genotypes of mice.

#### Barnes maze

For the Barnes maze spatial memory test, spatial cues were placed around the maze and these were kept constant throughout the study. For the baricitinib experiment, all mice from four experimental groups were consecutively subjected to the Barnes maze after 2 weeks, 6 weeks and 12 weeks of baricitinib or control treatment. For the CD8^+^ T cell depletion experiment, all mice from four experimental groups were subjected to the Barnes maze before and after 6-week anti-CD8 antibody or control treatment. The maze consists of a circular table with circular holes around its circumference. The goal is for the animal to reach the box that is positioned beneath one of the holes with the aid of visual cues. In the experiments, the surface of the table was brightly lit, serving as an aversive stimulus that motivated the mouse to find (and hide in) the goal box. The goal box location was the same in all testing runs. In the first 2 days of rehabilitation training, all mice were trained and guided by the same researcher to locate the box. Then, in the next 4 or 7 days, all mice learned where the box was located; the time needed to find the right aperture within the test duration of 180 s was automatically evaluated using video monitoring (EthoVision XT, Noldus Information Technology). To evaluate spatial memory, a spatial probe test was administered 24 h after the last training session. During the probe test, each mouse was allowed to search for the goal box on the platform for 180 s. Each test was terminated as soon as the mouse found the goal box or failed to find it within 180 s.

#### Open field test

The open field test is used to evaluate the ability for locomotion and exploration, and the level of anxiety in rodents. All mice were consecutively subjected to the open field test at different ages. During the open field test, mice were placed in a brightly illuminated transparent Plexiglas arena (50 × 50 × 30 cm) inside an enclosed cupboard. After 1 min of habituation, open field exploration was evaluated using video monitoring (EthoVision XT) for 15 or 30 min.

#### Elevated plus maze

The elevated plus maze is used to measure any differences in anxiety-related behaviors that can alter performance in other tests. All mice were consecutively subjected to the elevated plus maze at different ages. Briefly, the elevated plus maze has two arms enclosed by walls and two open arms without walls. Entries in both the closed and open arms, and the percentage of time spent in the open arms, were evaluated using video monitoring (EthoVision XT). Each mouse was placed in the left closed arm of the maze; after 1 min of habituation, exploratory activity was recorded for 5 min.

### Statistics

For the IHC analysis, 2–3 sections from each animal were analyzed. Data are shown as the mean ± s.e.m. Each dot represents one animal. The normal distribution of the samples was tested using the Shapiro–Wilk test. For the statistical analysis, a paired or unpaired Student’s *t*-test or Mann–Whitney *U*-test was used to compare two groups. A two-sided, one-way ANOVA followed by a Tukey’s post hoc test was used for multiple comparisons. Tests were chosen according to the distribution. In all tests, a *P* < 0.05 was considered significant, with *P < 0.05, **P < 0.01 and ***P < 0.001. The statistical analyses were done using Prism version 9.3.1 (GraphPad Software). Data acquisition and analysis were performed in a blinded manner. No animals were excluded from the analyses.

### Reporting summary

Further information on research design is available in the Nature Portfolio Reporting Summary linked to this article.

## Online content

Any methods, additional references, Nature Portfolio reporting summaries, source data, extended data, supplementary information, acknowledgements, peer review information; details of author contributions and competing interests; and statements of data and code availability are available at https://doi.org/10.1038/s41593-024-01682-8.

## Supplementary information

Supplementary Information Supplementary Figs. 1–7 and Tables 1 and 2.

Reporting Summary

Supplementary Tables 1 and 2 Information on the patients with AD (list of gene panels used for MERFISH).

Supplementary Data 1 Statistical source data for Supplementary Fig. 1.

Supplementary Data 2 Statistical source data for Supplementary Fig. 4.

Supplementary Data 3 Statistical source data for Supplementary Fig. 5.

Supplementary Data 4 Statistical source data for Supplementary Fig. 6.

## Source data

Source Data Fig. 1 Statistical source data.

Source Data Fig. 2 Statistical source data.

Source Data Fig. 3 Statistical source data.

Source Data Extended Data Fig. 1 Statistical source data.

Source Data Extended Data Fig. 2 Statistical source data.

Source Data Extended Data Fig. 3 Statistical source data.

Source Data Extended Data Fig. 4 Statistical source data.

Source Data Extended Data Fig. 5 Statistical source data.

Source Data Extended Data Fig. 6 Statistical source data.

Source Data Extended Data Fig. 7 Statistical source data.

Source Data Extended Data Fig. 8 Statistical source data.

Source Data Extended Data Fig. 9 Statistical source data.

Source Data Extended Data Fig.10 Statistical source data.

## Data Availability

The datasets we used (scRNA-seq and spatial transcriptomics) are deposited in the Gene Expression Omnibus database under accession nos GSE243018 and GSE243120. Source data are provided with this paper.

## Code availability

The code to analyze the scRNA-seq data of this manuscript is available on GitHub at https://github.com/Ruoqing-feng/AD_inflammation (ref. ^29^).

## References

[1] Prins, N. D. & Scheltens, P. White matter hyperintensities, cognitive impairment and dementia: an update. *Nat. Rev. Neurol.* **11**, 157–165 (2015). 10.1038/nrneurol.2015.10 25686760

[2] Kaya, T. et al. CD8^+^ T cells induce interferon-responsive oligodendrocytes and microglia in white matter aging. *Nat. Neurosci.* **25**, 1446–1457 (2022). 10.1038/s41593-022-01183-6 PMC9630119 36280798

[3] Mathys, H. et al. Single-cell transcriptomic analysis of Alzheimer’s disease. *Nature* **570**, 332–337 (2019). 10.1038/s41586-019-1195-2 PMC6865822 31042697

[4] Chen, W.-T. et al. Spatial transcriptomics and in situ sequencing to study Alzheimer’s disease. *Cell* **182**, 976–991 (2020). 10.1016/j.cell.2020.06.038 32702314

[5] Zhou, Y. et al. Human and mouse single-nucleus transcriptomics reveal TREM2-dependent and TREM2-independent cellular responses in Alzheimer’s disease. *Nat. Med.* **26**, 131–142 (2020). 10.1038/s41591-019-0695-9 PMC6980793 31932797

[6] Kenigsbuch, M. et al. A shared disease-associated oligodendrocyte signature among multiple CNS pathologies. *Nat. Neurosci.* **25**, 876–886 (2022). 10.1038/s41593-022-01104-7 PMC9724210 35760863

[7] Pandey, S. et al. Disease-associated oligodendrocyte responses across neurodegenerative diseases. *Cell Rep.* **40**, 111189 (2022). 10.1016/j.celrep.2022.111189 36001972

[8] Dean, D. C. 3rd et al. Association of amyloid pathology with myelin alteration in preclinical Alzheimer disease. *JAMA Neurol.* **74**, 41–49 (2017). 10.1001/jamaneurol.2016.3232 PMC5195903 27842175

[9] Falcão, A. M. et al. Disease-specific oligodendrocyte lineage cells arise in multiple sclerosis. *Nat. Med.* **24**, 1837–1844 (2018). 10.1038/s41591-018-0236-y PMC6544508 30420755

[10] Depp, C. et al. Myelin dysfunction drives amyloid-β deposition in models of Alzheimer’s disease. *Nature* **618**, 349–357 (2023). 10.1038/s41586-023-06120-6 PMC10247380 37258678

[11] Chen, J.-F. et al. Enhancing myelin renewal reverses cognitive dysfunction in a murine model of Alzheimer’s disease. *Neuron* **109**, 2292–2307 (2021). 10.1016/j.neuron.2021.05.012 PMC8298291 34102111

[12] Behrendt, G. et al. Dynamic changes in myelin aberrations and oligodendrocyte generation in chronic amyloidosis in mice and men. *Glia* **61**, 273–286 (2013). 10.1002/glia.22432 23090919

[13] Keren-Shaul, H. et al. A unique microglia type associated with restricting development of Alzheimer’s disease. *Cell* **169**, 1276–1290 (2017). 10.1016/j.cell.2017.05.018 28602351

[14] Sala Frigerio, C. et al. The major risk factors for Alzheimer’s disease: age, sex, and genes modulate the microglia response to Aβ plaques. *Cell Rep.* **27**, 1293–1306 (2019). 10.1016/j.celrep.2019.03.099 PMC7340153 31018141

[15] Yin, Z. et al. Identification of a protective microglial state mediated by miR-155 and interferon-γ signaling in a mouse model of Alzheimer’s disease. *Nat. Neurosci.* **26**, 1196–1207 (2023). 10.1038/s41593-023-01355-y PMC10619638 37291336

[16] Krasemann, S. et al. The TREM2-APOE pathway drives the transcriptional phenotype of dysfunctional microglia in neurodegenerative diseases. *Immunity* **47**, 566–581 (2017). 10.1016/j.immuni.2017.08.008 PMC5719893 28930663

[17] Safaiyan, S. et al. White matter aging drives microglial diversity. *Neuron* **109**, 1100–1117 (2021). 10.1016/j.neuron.2021.01.027 33606969

[18] Ting, J. P. & Baldwin, A. S. Regulation of MHC gene expression. *Curr. Opin. Immunol.* **5**, 8–16 (1993). 10.1016/0952-7915(93)90074-3 8452678

[19] Jorfi, M. et al. Infiltrating CD8^+^ T cells exacerbate Alzheimer’s disease pathology in a 3D human neuroimmune axis model. *Nat. Neurosci.* **26**, 1489–1504 (2023). 10.1038/s41593-023-01415-3 PMC11184920 37620442

[20] Chen, X. et al. Microglia-mediated T cell infiltration drives neurodegeneration in tauopathy. *Nature* **615**, 668–677 (2023). 10.1038/s41586-023-05788-0 PMC10258627 36890231

[21] Oakley, H. et al. Intraneuronal β-amyloid aggregates, neurodegeneration, and neuron loss in transgenic mice with five familial Alzheimer’s disease mutations: potential factors in amyloid plaque formation. *J. Neurosci.* **26**, 10129–10140 (2006). 10.1523/JNEUROSCI.1202-06.2006 PMC6674618 17021169

[22] Saito, T. et al. Single App knock-in mouse models of Alzheimer’s disease. *Nat. Neurosci.* **17**, 661–663 (2014). 10.1038/nn.3697 24728269

[23] Mombaerts, P. et al. RAG-1-deficient mice have no mature B and T lymphocytes. *Cell* **68**, 869–877 (1992). 10.1016/0092-8674(92)90030-g 1547488

[24] Hyman, B. T. et al. National Institute on Aging-Alzheimer’s Association guidelines for the neuropathologic assessment of Alzheimer’s disease. *Alzheimers Dement.* **8**, 1–13 (2012). 10.1016/j.jalz.2011.10.007 PMC3266529 22265587

[25] Colombo, A. et al. Loss of NPC1 enhances phagocytic uptake and impairs lipid trafficking in microglia. *Nat. Commun.* **12**, 1158 (2021). 10.1038/s41467-021-21428-5 PMC7904859 33627648

[26] Kislinger, G. et al. Multiscale ATUM-FIB microscopy enables targeted ultrastructural analysis at isotropic resolution. *iScience* **23**, 101290 (2020). 10.1016/j.isci.2020.101290 PMC7334410 32622266

[27] Schindelin, J. et al. Fiji: an open-source platform for biological-image analysis. *Nat. Methods* **9**, 676–682 (2012). 10.1038/nmeth.2019 PMC3855844 22743772

[28] Liu, L. et al. Dissociation of microdissected mouse brain tissue for artifact free single-cell RNA sequencing. *STAR Protoc.* **2**, 100590 (2021). 10.1016/j.xpro.2021.100590 PMC8196224 34159323

[29] Kedia, S., Feng, R., & Simons, M. T cell-mediated microglia activation triggers myelin pathology in a mouse model of amyloidosis. *Zenodo*10.5281/zenodo.11189582 (2024). PMC11303250 38937583

