# Author Correction: T cell-mediated microglial activation triggers myelin pathology in a mouse model of amyloidosis

**DOI:** 10.1038/s41593-026-02502-x

**Published:** 2026-10-13

**Authors:** Shreeya Kedia, Hao Ji, Ruoqing Feng, Peter Androvic, Lena Spieth, Lu Liu, Jonas Franz, Hanna Zdiarstek, Katrin Perez Anderson, Cem Kaboglu, Qian Liu, Nicola Mattugini, Fatma Cherif, Danilo Prtvar, Ludovico Cantuti-Castelvetri, Arthur Liesz, Martina Schifferer, Christine Stadelmann, Sabina Tahirovic, Ozgun Gokce, Mikael Simons

**Affiliations:** 1 Institute of Neuronal Cell Biology, Technical University Munich, Munich, Germany; 2 German Center for Neurodegenerative Diseases (DZNE), Munich, Germany; 3 Institute for Stroke and Dementia Research, University Hospital of Munich, LMU Munich, Munich, Germany; 4 Department of Neurodegenerative Diseases and Geriatric Psychiatry, University Hospital Bonn, Bonn, Germany; 5 Department of Neuropathology, University Medical Center Göttingen, Göttingen, Germany; 6 Munich Cluster of Systems Neurology (SyNergy), Munich, Germany; 7 German Center for Neurodegenerative Diseases (DZNE), Bonn, Germany

**Keywords:** Glial biology, Experimental models of disease

Correction to: *Nature Neuroscience* https://doi.org/10.1038/s41593-024-01682-8, published online 27 June 2024.

In the version of this article initially published, the Source Data for Fig. 1h and Extended Data Fig. 10b contained values from an internal working file used to validate tracking rather than the final analysis. In addition, the Source Data files for Extended Data Figs. 2–7 retained figure labels from an earlier version of the manuscript. The affected Source Data files have been replaced, Fig. 1h has been updated, the figure labels have been corrected, and the legend of Extended Data Fig. 10 has been revised to report the correct sample sizes. These corrections do not affect the conclusions of the study. For comparison, the original Fig. 1 is available as Supplementary Information accompanying this amendment.

## Supplementary information

Original Fig. 1

