# Supplementary material for: T cell-mediated microglial activation triggers myelin pathology in a mouse model of amyloidosis
Source: Nat Neurosci. 2024 Jun 27;27(8):1468–74. doi: 10.1038/s41593-024-01682-8 (PMC11303250; doi:10.1038/s41593-024-01682-8)
Supplement: Supplementary file 1 — Supplementary Figs. 1–7 and Tables 1 and 2. [file 41593_2024_1682_MOESM1_ESM.pdf]

nature neuroscience

Supplementary information

https://doi.org/10.1038/s41593-024-01682-8

T cell-mediated microglial activation triggers myelin pathology in a mouse model of amyloidosis

In the format provided by the authors and unedited

SUPPLEMENTARY FIGURES

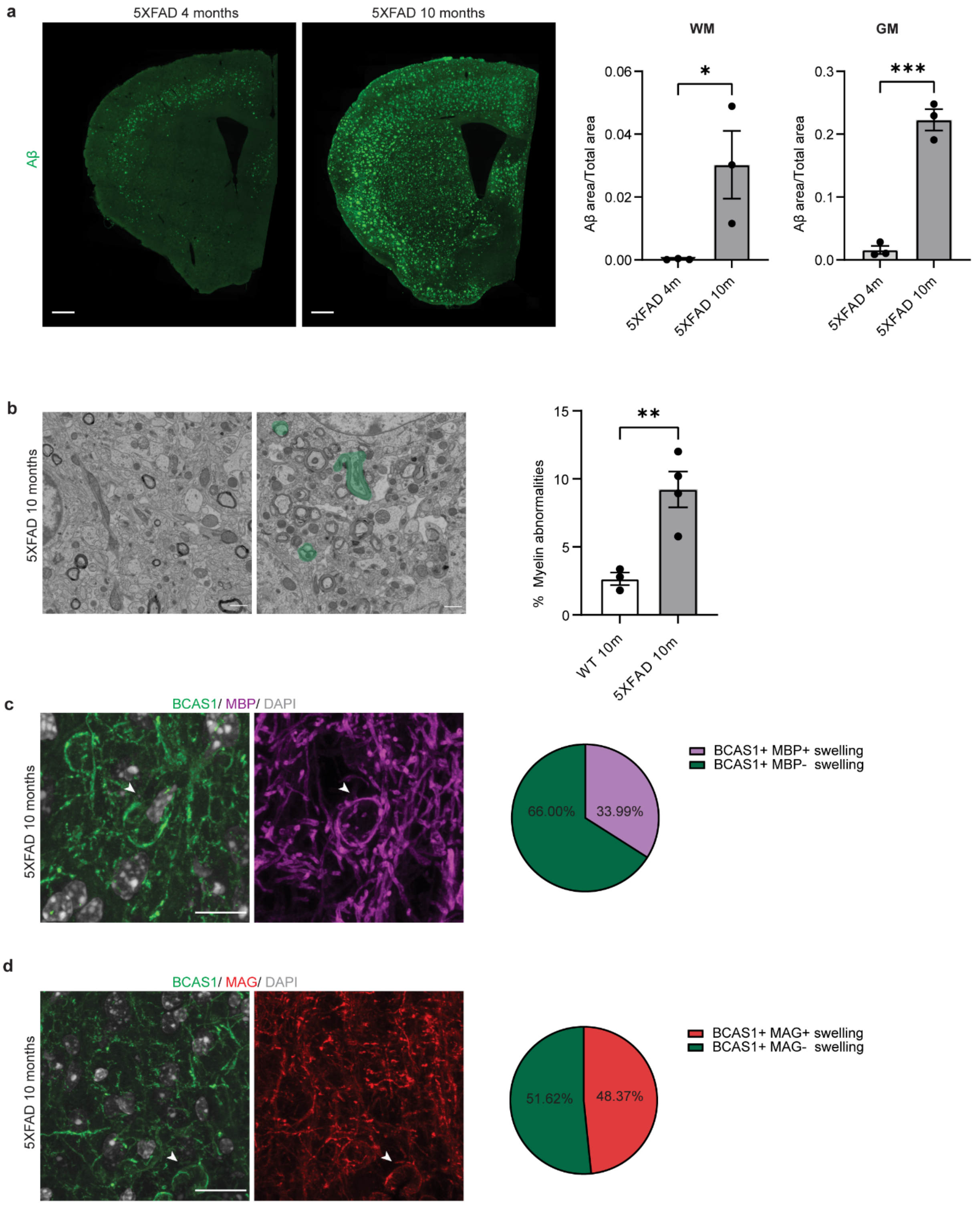

Supplementary Figure 1 | Evidence of increased oligodendrocyte and myelin damage in *5xFAD* mice. a, Representative image showing Aβ plaque load (green) in the cortex and corpus callosum of *5xFAD* mice aged 4 and 10 months. Scale bar, 500 μm. Quantifications indicate proportion of Aβ positive area normalized to total area in the cortex. Statistical significance was determined for n=3 animals using unpaired two-sided Student's t-test (WM, *5xFAD* 4m, *5xFAD* 10m, *p=0.04; GM, *5xFAD* 4m, *5xFAD* 10m, ***p=0.0003). **b,** Representative scanning electron microscopy images of the cortex of female WT and *5xFAD* mice aged 10 months. Myelin abnormalities are shown in green. Scale bar, 1 μm. Quantifications indicate total percentage of myelinated axons with myelin abnormalities. Statistical significance was determined for n=3 animals using unpaired two-sided Student's t-test (WT 10m, *5xFAD* 10m, **p=0.0093). **c,** Representative image of BCAS1^{+} (green) MBP^{+} (magenta) swellings lacking a nucleus (stained with DAPI). Scale bar, 10 μm. Pie chart represents mean percentage of BCAS1^{+} swellings positive or negative for MBP in n=3 animals. **d,** Representative image of BCAS1^{+} (green) MAG^{+} (red) swellings lacking a nucleus (stained with DAPI). Scale bar, 10 μm. Pie chart represents mean percentage of BCAS1^{+} swellings positive or negative for MAG in n=3 animals. Each point on the graph represents one animal. Data is presented as mean ± SEM.

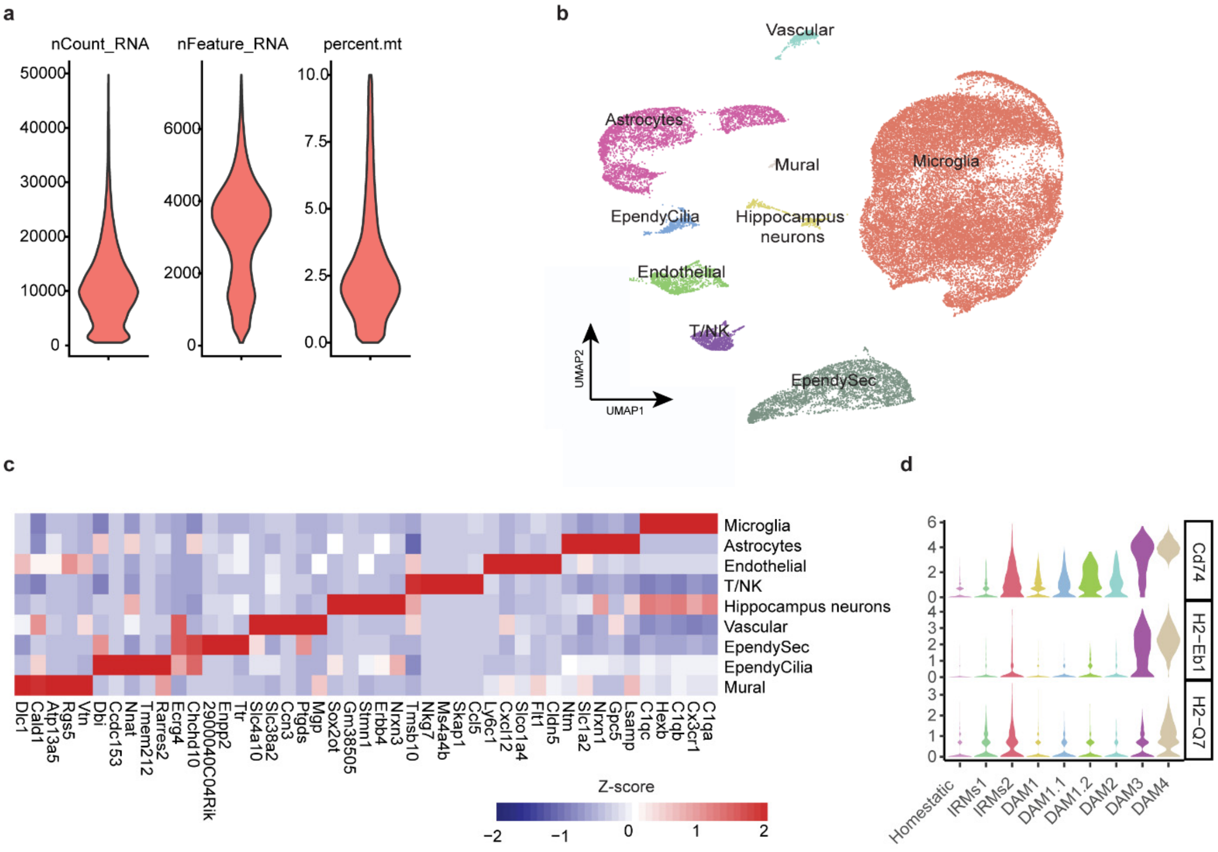

Supplementary Figure 2 | Quality control of sc-RNA sequencing data. a, Violin plots showing single cell total UMI count, detected RNA count, and mitochondria UMI proportion distribution of the dataset. **b,** UMAP plots of all detected single cells, colored by identified clusters. **c,** Heatmaps of scaled average expression of selected marker genes of all single cell clusters. Each column represents a gene. **d,** Violin plots showing expression of selected MHCII genes in all microglia populations.

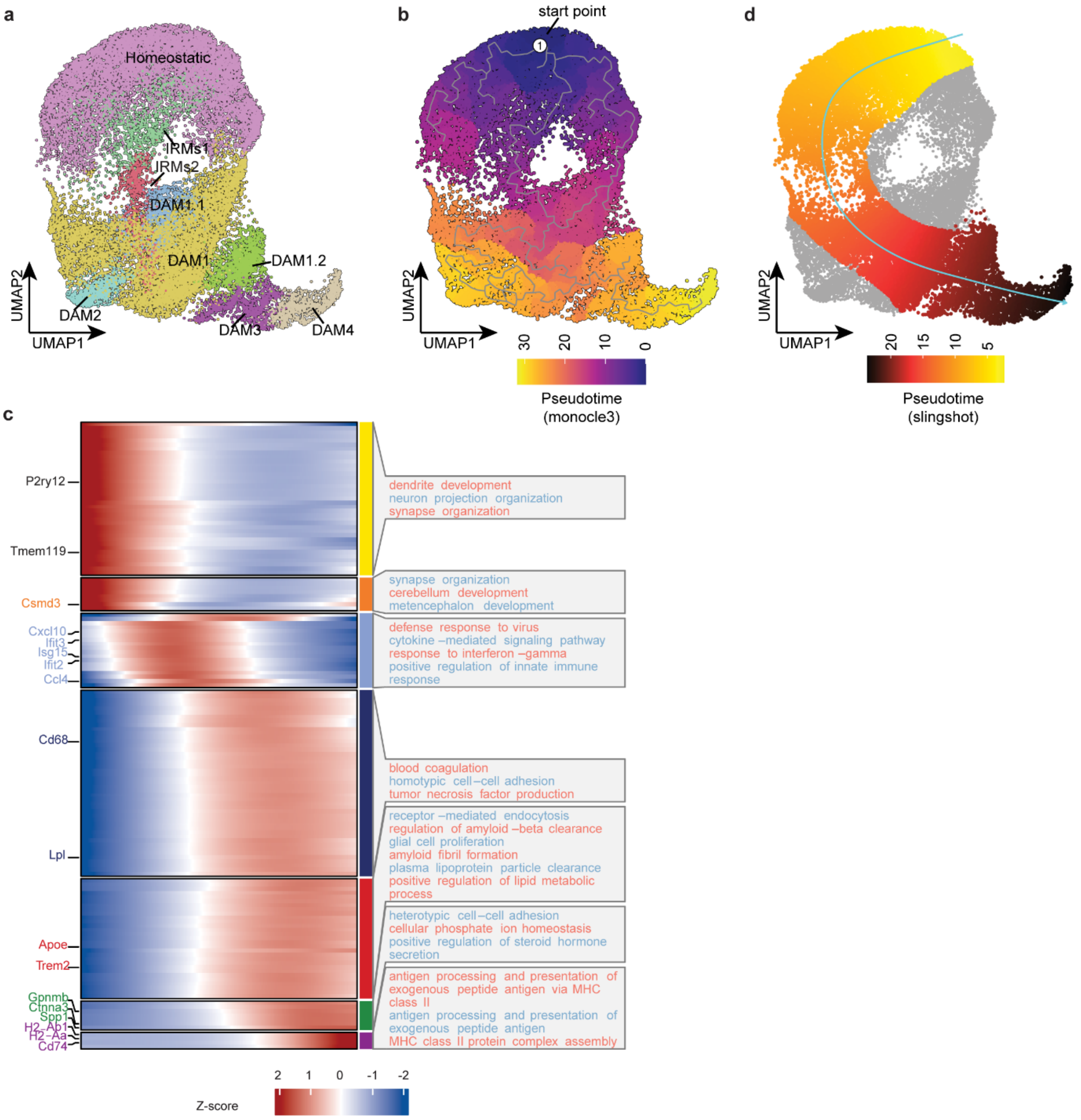

Supplementary Figure 3 | Trajectory analysis showing transition from homeostatic to disease-associated microglia 4 (DAM4) cluster. a, UMAP plot of microglia, colored by identified populations. **b**, UMAP plot of microglia, colored by pseudotime (calculated by monocle3). **c,** Heatmap of scaled gene expression of microglia on the monocle3 trajectory from homeostatic to DAM4. The highly variable genes over the trajectory were selected by monocle3 (see Methods). All single cell on the trajectory were ordered by pseudotime (monocle3) from smallest to largest. All the genes were clustered into 7 modules. GO terms are shown on the right of genes. Only biology processed (BP) terms were involved. **d,** UMAP plot of microglia, colored by pseudotime (calculated by slingshot).

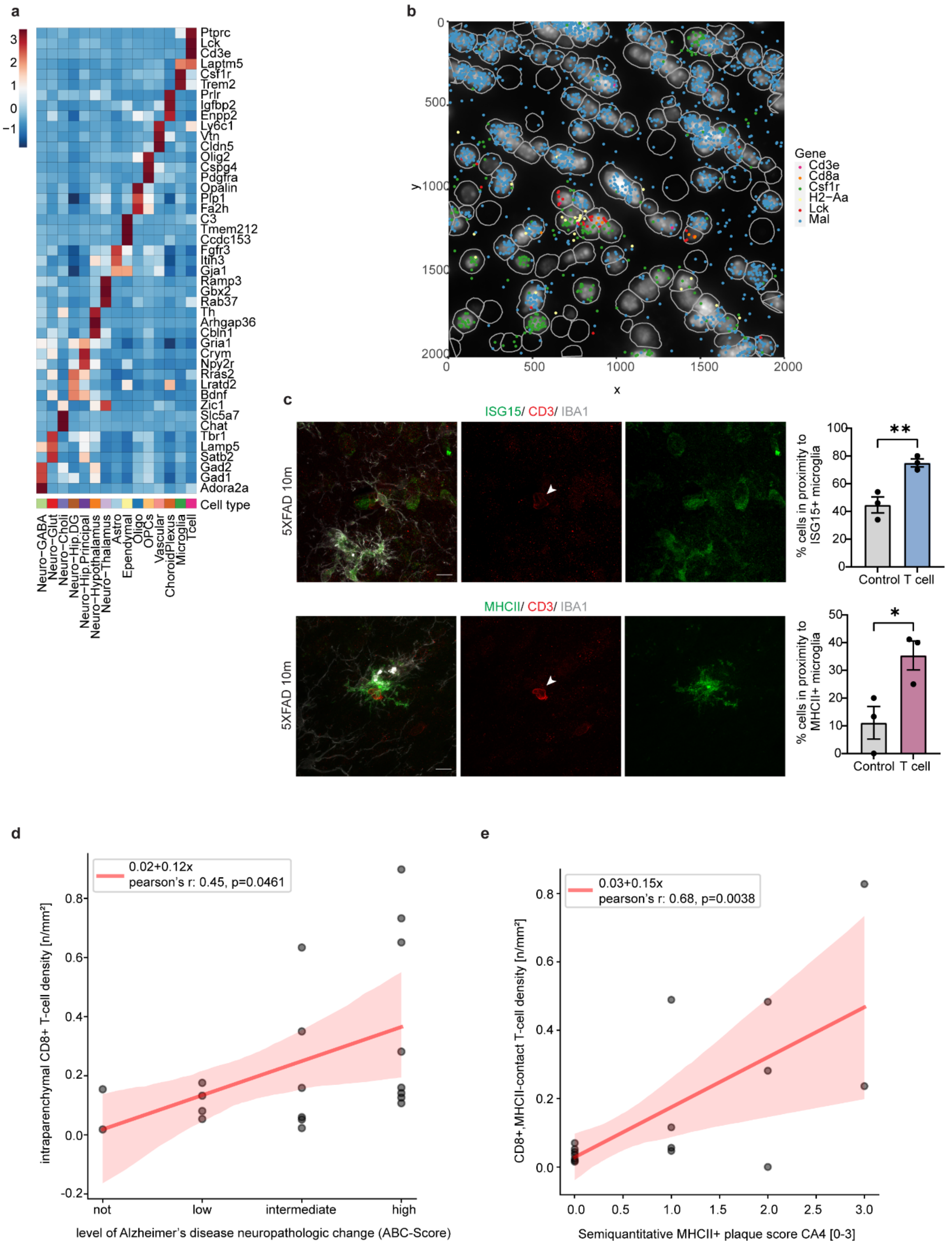
a
3
2
1
0
−1
Ptprc
Lck
Cd3e
Laptm5
Csf1r
Trem2
Prlr
Igfbp2
Enpp2
Ly6c1
Vtn
Cldn5
Olig2
Cspg4
Pdgfra
Opalin
Plp1
Fa2h
C3
Tmem212
Ccdc153
Fgfr3
Itih3
Gja1
Ramp3
Gbx2
Rab37
Th
Arhgap36
Cbln1
Gria1
Crym
Npy2r
Rras2
Lratd2
Bdnf
Zic1
Slc5a7
Chat
Tbr1
Lamp5
Satb2
Gad2
Gad1
Adora2a
Cell type
Neuro−GABA
Neuro−Glut
Neuro−Choli
Neuro−Hip.DG
Neuro−Hip.Principal
Neuro−Hypothalamus
Neuro−Thalamus
Astro
Ependymal
Oligo
OPCs
Vascular
ChoroidPlexus
Microglia
Tcell
b
Gene
Cd3e
Cd8a
Csf1r
H2−Aa
Lck
Mal
x
y
c
ISG15/ CD3/ IBA1
5XFAD 10m
% cells in proximity to ISG15+ microglia
Control
T cell
MHCII/ CD3/ IBA1
% cells in proximity to MHCII+ microglia
d
0.02+0.12x
pearson's r: 0.45, p=0.0461
intraparenchymal CD8+ T-cell density [n/mm²]
not
low
intermediate
high
level of Alzheimer's disease neuropathologic change (ABC-Score)
e
0.03+0.15x
pearson's r: 0.68, p=0.0038
CD8+,MHCII-contact T-cell density [n/mm²]
Semiquantitative MHCII+ plaque score CA4 [0-3]

Supplementary Figure 4 | Analysis of CD8$^+$ microglia activation using MERFISH spatial transcriptomics. a, Heatmap showing average expression of cell type markers in identified cell populations. **b,** Spatial location of individual transcripts of selected marker genes for CD8$^+$ T-cells (Cd3e, Cd8a, Lck), microglia (Csf1r), oligodendrocytes (Mal) and MHCII-complex members (H2-Aa) superimposed over DAPI signal. Representative image from 9.5 month-old 5xFAD brain section is shown. **c**, Representative image (upper panel) showing ISG15$^+$ (green) IBA1$^+$ (grey) cell and CD3$^+$ T cell (red) in *5xFAD* mice aged 10 months. Scale bar, 10 µm. Quantifications indicate the percentage of T cells in proximity to ISG15$^+$ IBA1$^+$ cells in comparison to a random DAPI$^+$ cell (Control). Statistical significance was determined for n=3 animals using unpaired two-sided Student's t-test (Control, T cell **p=0.009). Representative image (lower panel) showing MHCII$^+$ (green) IBA1$^+$ (grey) cell and CD3$^+$ T cell (red) in *5xFAD* mice aged 10 months. Quantifications indicate the percentage of T cells in proximity to MHCII$^+$ IBA1$^+$ cells in comparison to a random DAPI$^+$ cell (Control). Statistical significance was determined using unpaired two-sided Student's t-test (Control, T cell *p=0.03). Each point on the graph represents one animal. Data is presented as mean ± SEM. **d**, Level of neuropathological AD probability according to the NIH criteria (ABC score; not, low, intermediate, high) correlate significantly with CD8$^+$ T cell density in the CA4 region of the hippocampus. (Pearson's r: 0.45, p=0.0461). A linear regression model revealed the interaction with 0.02 + 0.12x and is displayed in red with 95% confidence intervals. Visualized is a linear regression line with 95% confidence intervals from a distribution of n_boot=1000 resamples. **e,** MHCII$^+$ amyloid plaque density measured semiquantiatively in the CA4 region of the hippocampus correlate significantly with CD8$^+$ T cell density in contact with MHCII$^+$ cells (Pearson's r: 0.68, p=0.0038). A linear regression model revealed the interaction with 0.03 + 0.15x and is displayed in red with 95% confidence intervals. Visualized is a linear regression line with 95% confidence intervals from a distribution of n_boot=1000 resamples.

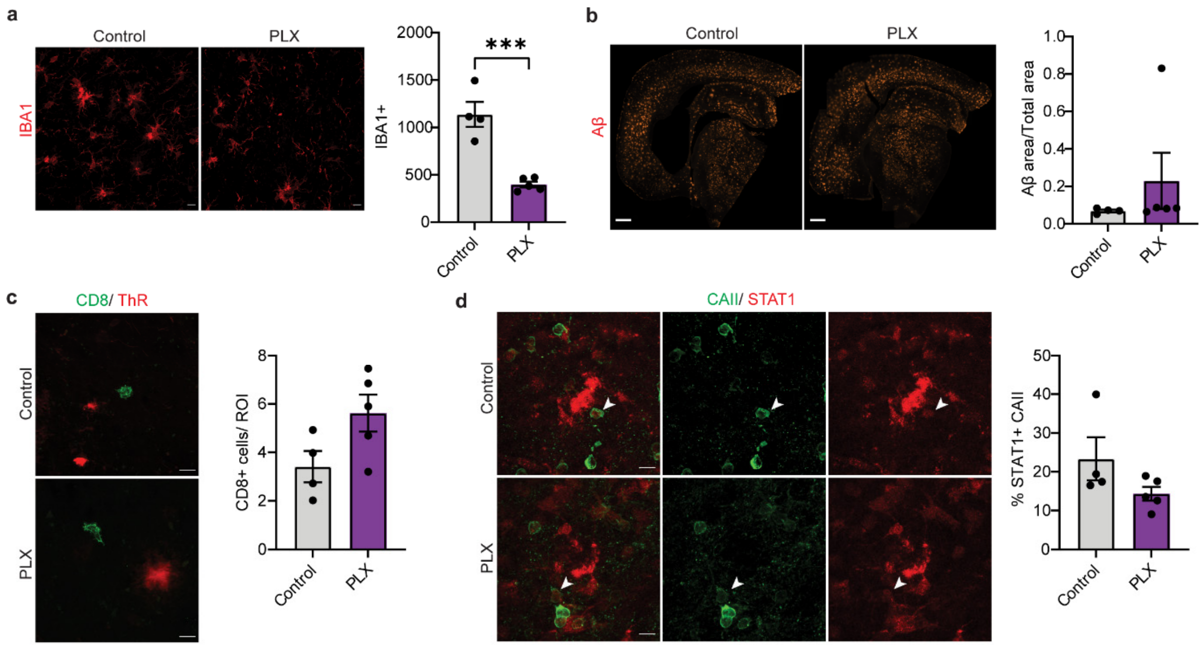

Supplementary Figure 5 | Microglia depletion reduces oligodendrocyte and myelin damage in *5xFAD* mice. a, Representative image showing IBA1^{+} cells (red) in the cortex of 7.5-months-old *5xFAD* mice treated with either control or PLX5622 (PLX) chow. Scale bar, 10 µm. Quantifications indicate the number of IBA1^{+} cells normalized to total area in the cortex. Statistical significance was determined for n=4-5 animals using unpaired two-sided Student's t-test (Control, PLX, ***p=0.0005). **b,** Representative image showing Aβ plaque load (red) in 7.5 months old *5xFAD* mice treated with either control of PLX chow. Scale bar, 500 µm. Quantifications indicate proportion of Aβ positive area normalized to total area in the cortex. Statistical significance was determined for n=4-5 animals using unpaired Student's t-test. **c,** Representative image showing CD8^{+} T cells (green) in the cortex of 7.5-months-old *5xFAD* mice treated with either control or PLX chow. Plaques are stained with Thiazine Red (ThR, red). Scale bar, 10 µm. Quantifications indicate the number of CD8^{+} T cells in the cortex. Statistical significance was determined for n=4-5 animals using unpaired Student's t-test. **d,** Representative image showing STAT1^{+} (red) CAII^{+} (green) oligodendrocytes in the cortex of 7.5 month-old *5xFAD* mice treated with either control or PLX chow. Scale bar, 10 µm. Quantifications indicate the percentage of CAII^{+} cells also positive for STAT1. Statistical significance was determined for n=4-5 animals using unpaired Student's t-test. Each point on the graph represents one animal. Data is presented as mean ± SEM.

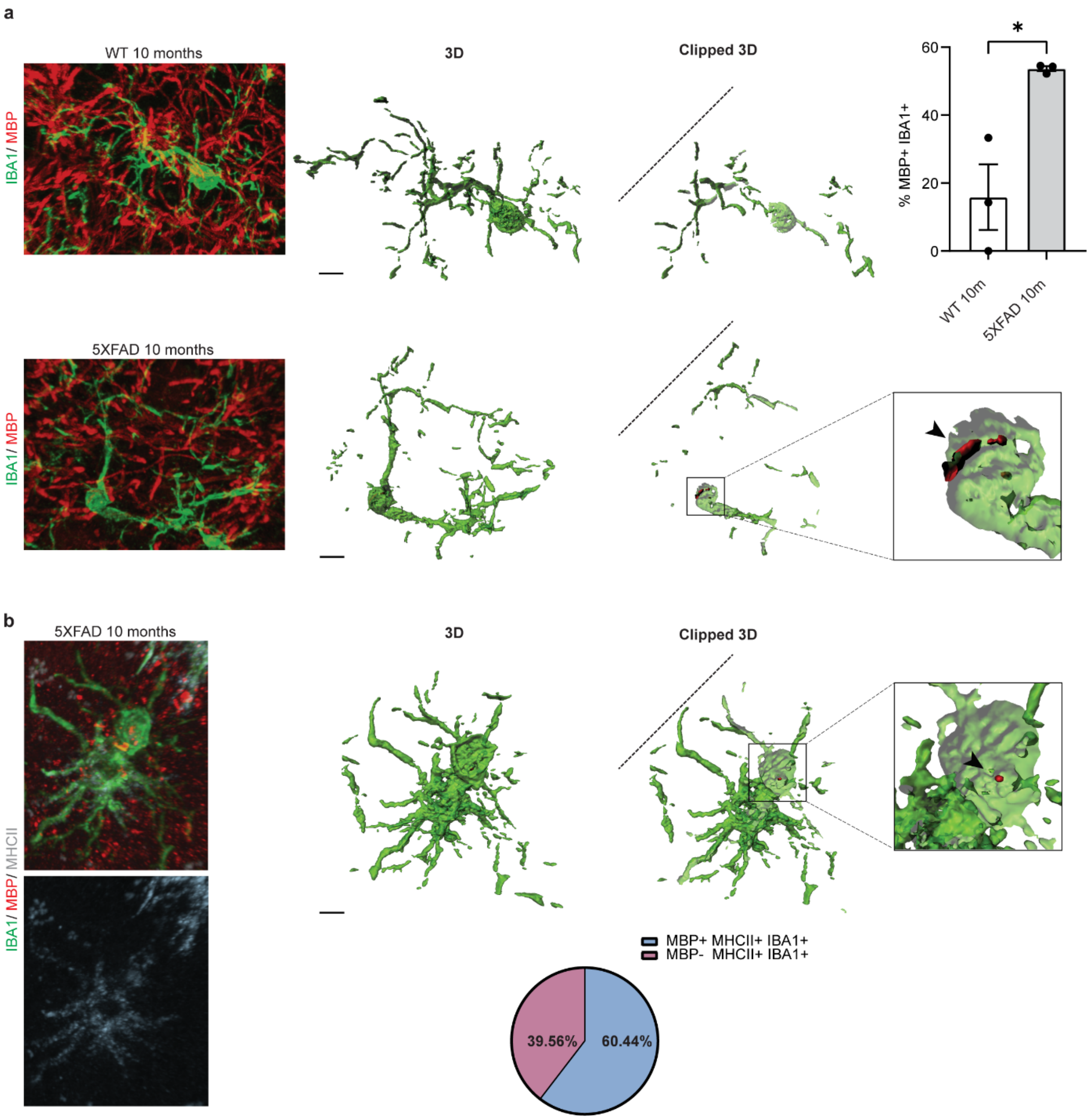

Supplementary Figure 6 | Presence of internalized myelin fragments in microglia of *5xFAD* mice. a, Representative immunofluorescence image (left) showing IBA1$^+$ cells (green) with and without internalized MBP (red) in the cortex of *5xFAD* and WT mice aged 10 months respectively. 3D (middle), clipped 3D (middle) and magnified clipped 3D (right) image showing internalized MBP in IBA1$^+$ cells in *5xFAD* mice. Arrowheads show MBP. Scale bar, 5 µm. Quantifications indicate the percentage of IBA1$^+$ cells also positive for MBP. Statistical significance was determined for n=3 animals using unpaired two-sided Student's t-test (WT 10m, *5xFAD* 10m, *p=0.017). Each point on the graph represents one animal. Data is presented as mean ± SEM. **b,** Representative immunofluorescence image (upper panel) showing MHCII$^+$ (grey) IBA1$^+$ cells (green) containing internalized MBP (red) in *5xFAD* mice aged 10 months. 3D (lower panel, left), clipped 3D (lower panel,middle) and magnified clipped 3D (lower panel, right) image showing internalized MBP in MHCII$^+$ IBA1$^+$ cells. Arrowheads show MBP. Scale

bar, 3 μm. Pie chart indicates mean percentage of $MHCII^+$ $IBA1^+$ cells positive for MBP in n=3 animals.

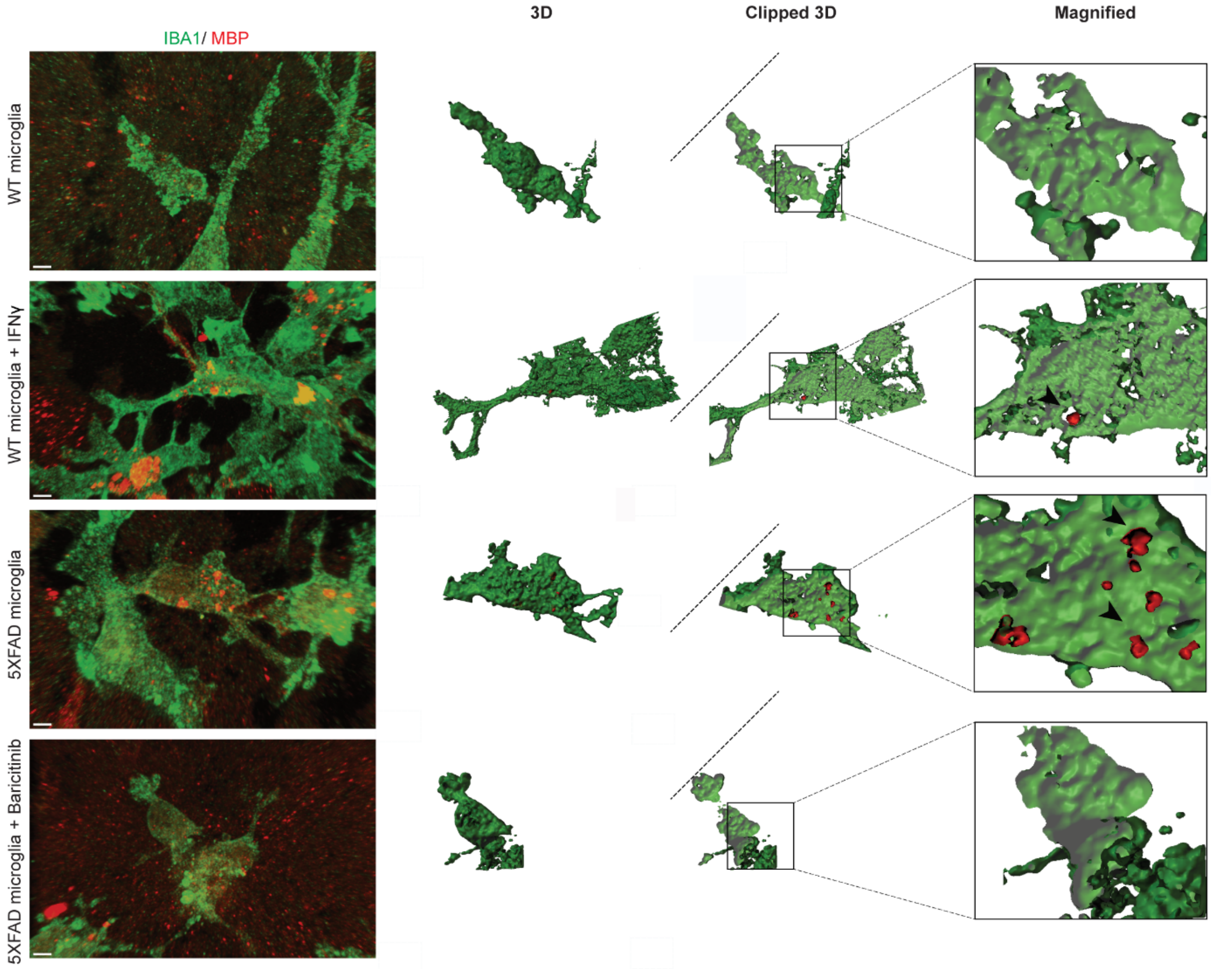

Supplementary Figure 7 | Evidence of internalized MBP+ material in activated microglia in an *ex-vivo* slice culture model. Representative immunofluorescence image from n=3-5 independent experiments (left), 3D (middle), clipped 3D (middle) and magnified clipped 3D (right) showing IBA1^{+} cells (green) with internalized MBP (red) in untreated WT microglia, WT microglia treated with IFNγ, untreated *5xFAD* microglia and *5xFAD* microglia treated with baricitinib. Arrowheads show MBP. Scale bar, 4 µm.

Supplementary Table 1 | Information on AD patients. A list of gene panel used for MERFISH.
