# Supplementary material for: T cell-mediated microglial activation triggers myelin pathology in a mouse model of amyloidosis
Source: Nat Neurosci. 2024 Jun 27;27(8):1468–74. doi: 10.1038/s41593-024-01682-8 (PMC11303250; doi:10.1038/s41593-024-01682-8)
Supplement: Supplementary file 2 — Reporting Summary [file 41593_2024_1682_MOESM2_ESM.pdf]

nature portfolio

Corresponding author(s): Mikael Simons

Last updated by author(s): Aug 30, 2023

Reporting Summary

Nature Portfolio wishes to improve the reproducibility of the work that we publish. This form provides structure for consistency and transparency in reporting. For further information on Nature Portfolio policies, see our Editorial Policies and the Editorial Policy Checklist.

Statistics

For all statistical analyses, confirm that the following items are present in the figure legend, table legend, main text, or Methods section.

n/a	Confirmed	
☐	☒	The exact sample size (*n*) for each experimental group/condition, given as a discrete number and unit of measurement
☐	☒	A statement on whether measurements were taken from distinct samples or whether the same sample was measured repeatedly
☐	☒	The statistical test(s) used AND whether they are one- or two-sided<br>*Only common tests should be described solely by name; describe more complex techniques in the Methods section.*
☒	☐	A description of all covariates tested
☐	☒	A description of any assumptions or corrections, such as tests of normality and adjustment for multiple comparisons
☐	☒	A full description of the statistical parameters including central tendency (e.g. means) or other basic estimates (e.g. regression coefficient) AND variation (e.g. standard deviation) or associated estimates of uncertainty (e.g. confidence intervals)
☐	☒	For null hypothesis testing, the test statistic (e.g. *F*, *t*, *r*) with confidence intervals, effect sizes, degrees of freedom and *P* value noted<br>*Give P values as exact values whenever suitable.*
☒	☐	For Bayesian analysis, information on the choice of priors and Markov chain Monte Carlo settings
☒	☐	For hierarchical and complex designs, identification of the appropriate level for tests and full reporting of outcomes
☐	☒	Estimates of effect sizes (e.g. Cohen's *d*, Pearson's *r*), indicating how they were calculated

Our web collection on statistics for biologists contains articles on many of the points above.

Software and code

Policy information about availability of computer code

Data collection

1. Confocal images: all images of mouse brain sections were acquired using a Leica TCS SP5 or Zeiss LSM900 confocal microscope.

2. Electron microscopy: Scanning Electron Microscope images were acquired on a Crossbeam Gemini 34 SEM (Zeiss) with a four-quadrant backscatter detector at 8kV using ATLAS5 Array Tomography (Fibics). Medium lateral resolution images (100 nm) allowed the identification of regions of interest that were in turn reimaged at 4-10 nm lateral resolution.

3. Single cell RNA-seq: Animals were flushed with PBS and brains were microdissected under a dissection micrscope. Cells were isolated using a previously described protocol using gentleMACS with Neural Tissue Dissociation Kit and actinomycinD. Myelin debris was removed using Myelin Removal Beads II (Miltenyi Biotec). Cells were counted using TC20 Bio-Rad before loading to Chromium Controller.

4. MERFISH: Vizgen microscope system was used for image acquisition.

Data analysis

1. Confocal images: all the image analysis and image processing were done using Imaris (64x version 9.2.0) and ImageJ 1.41 image processing softwares.

2. Electron microscopy images: Image analysis was performed in Fiji.

3. Single cell RNA-seq data analysis: The raw sequencing data of each library was processed by Cell Ranger (10X Genomics) 3.0 to generate the gene-by-cell unique molecular identifier (UMI) count matrix. The single cell gene expression matrix was imported into R package Seurat (4.3.0). For further analysis, cells were excluded according to total UMI count (less or equal than 50000); total detected gene number (less or equal than 7500); and mitochondrial UMI percentage (less than 10%). To prevent the influence of technical characteristics of downstream analyses, we performed the SCTransform to normalize the clean UMI matrix. The single cell clustering was done according to the vignette (https://satijalab.org/seurat/articles/sctransform_v2_v
ignette.html). The variable features were selected with residual variance more than 1.4, the principal component analysis (PCA) was

performed to normalized matrix. The cluster markers were identified by the FindAllMarkers function. Microglia subclusters were identified using the same parameters described above. Microglia subcluster compositional analysis was done by scCODA v.0.1.9 according to the online vignette (https://sccoda.readthedocs.io/en/latest/getting_started.html). To capture nuanced yet biologically significant alterations, the false discovery rate (FDR) was established at 0.4, as described in above vignette. The code to analyze the scRNA-seq data of this manuscript is available on GitHub: https://github.com/Ruoqing-feng/AD_inflammation.

4. Single cell trajectory construction: The clean UMI matrix of all microglia were imported to R package Monocle3 and processed with normalization and PCA analysis. Functions learn_graph and order_cells were used to construct the trajectory. Homeostatic microglia were set as the start point of the trajectory. The function choose_graph_segments was used to determine the path with homeostatic microglia as the start point and DAM4 as the end point. Moran's I test was performed in function graph_test to find differentially expressed genes (q value less than 0.05 and Moran's index more than 0.3) across the selected path. For Slingshot trajectory analysis, the clean UMI matrix of all microglia was imported to R package SingleCellExperiment and processed with normalization and PCA analysis. Function slingshot was used to construct the trajectory without an indicated start or end point.

5. MERFISH: Raw images were decoded to RNA spots with spatial coordinates and gene id using Vizgen's Merlin software on the Merscope instrument. Cell segmentation was performed using CellPose algorithm, using DAPI nuclear and PolyT total RNA staining channels. Resulting single cell gene expression matrices were further analyzed in R.

4. Statistical analyses were done using GraphPad Prism 9.3.1 (GraphPad Software, Inc.).

For manuscripts utilizing custom algorithms or software that are central to the research but not yet described in published literature, software must be made available to editors and reviewers. We strongly encourage code deposition in a community repository (e.g. GitHub). See the Nature Portfolio guidelines for submitting code & software for further information.

Data

Policy information about availability of data

All manuscripts must include a data availability statement. This statement should provide the following information, where applicable:

- Accession codes, unique identifiers, or web links for publicly available datasets
- A description of any restrictions on data availability
- For clinical datasets or third party data, please ensure that the statement adheres to our policy

The data supporting the findings of this study are available in a publicly available repository.

Field-specific reporting

Please select the one below that is the best fit for your research. If you are not sure, read the appropriate sections before making your selection.

☒ Life sciences ☐ Behavioural & social sciences ☐ Ecological, evolutionary & environmental sciences

For a reference copy of the document with all sections, see nature.com/documents/nr-reporting-summary-flat.pdf

Life sciences study design

All studies must disclose on these points even when the disclosure is negative.

Sample size	No power analyses were used to predetermine sample sizes. However, sample sizes were chosen based on prior literature using similar experimental paradigms.
Data exclusions	No data were excluded from analysis.
Replication	For all mouse experiments, 3-6 independent biological replicates per condition were processed and replicated independently. We confirm that all independent attempts were successful.
Randomization	The allocation of samples including brain sections was random.
Blinding	All data acquisition and analyses was done blinded. Except for the electron microscopy analyses of wild-type and 5XFAD, the plaques were visible, leading to unblinding.

Reporting for specific materials, systems and methods

We require information from authors about some types of materials, experimental systems and methods used in many studies. Here, indicate whether each material, system or method listed is relevant to your study. If you are not sure if a list item applies to your research, read the appropriate section before selecting a response.

Materials & experimental systems

n/a	Involved in the study
☐	☒ Antibodies
☒	☐ Eukaryotic cell lines
☒	☐ Palaeontology and archaeology
☐	☒ Animals and other organisms
☐	☒ Human research participants
☒	☐ Clinical data
☒	☐ Dual use research of concern

Methods

n/a	Involved in the study
☒	☐ ChIP-seq
☒	☐ Flow cytometry
☒	☐ MRI-based neuroimaging

Antibodies

Antibodies used

1. mouse anti-APC clone CC1(Millipore, OP80-100UG, 1:100)
2. rabbit anti-B2m (abcam, ab75853-100ul, 1:100)
3. rabbit anti-STAT1 (Cell Signaling Technology, 14994S, 1:100)
4. rat anti-CD8 (Biolegend, 100702, 1:100)
5. rabbit anti-Iba1 (Wako, 234 004, 1:250)
6. chicken anti-Iba1 (Synaptic Systems, 234009, 1:400)
7. mouse anti-β-Amyloid1-11 (Biolegend, 835104, 1:1000)
8. goat anti-Serpina3n (Bio-Techne, AF4709, 1:100)
9. rabbit anti-Olig2 (Millipore, AB9610, 1:250)
10. anti-PDGF-Ralpha (R&D Systems, AF1062 1:100)
11. rat anti-MHCII (Invitrogen, 14-5321-82, 1:100)
12. mouse anti-ISG15 (F-9) (Santa Cruz, sc-166755, 1:100)
13. rat anti-MBP (Abcam, ab7349, 1:300)
14. chicken anti-MBP (Invitrogen, PA1-10008, 1:400)
15. rabbit anti-BCAS1 (generated against amino acids 1-667 of mouse BCAS1, custom-made by Synaptic Systems, Göttingen, 1:500),
16. mouse anti-MAG clone 513 (Millipore, MAB1567, 1:200)

1. anti-mouse 488 (Thermo Fisher Scientific, A-21202, 1:500)
2. anti-mouse 555 (Thermo Fisher Scientific, A-21422, 1:500)
3. anti-mouse 647 (Thermo Fisher Scientific, A-21235, 1:500)
4. anti-rabbit 488 (Thermo Fisher Scientific, A-11008,1:500)
5. anti-rabbit 555 (Thermo Fisher Scientific, A-21428, 1:500)
6. anti-rat 555 (Thermo Fisher Scientific, A-21434, 1:500)
7. anti-goat 555 (Thermo Fisher Scientific, A-21432, 1:500)
8. anti-goat 647 (Thermo Fisher Scientific, A-32849, 1:500)
9. donkey anti-rat 488 (Thermo Fisher Scientific, A-21208, 1:500)
10. donkey anti-rabbit 647 (Thermo Fisher Scientific, A-31573, 1:500)
11. donkey anti-goat 555 (Thermo Fisher Scientific, A-32816, 1:500)

InVivoPlus anti-mouse CD8 antibody, BioXCell, BP0061
InVivoPlus rat IgG2b isotype control, anti-keyhole limpet hemocyanin, BioXCell, BP0090
InVivoPlus anti-mouse PD-1, BioXCell, BP0146
InVivoPlus anti-mouse CTLA-4, BioXCell, BP0131
InVivoMAb rat IgG2a isotype control, anti-trinitrophenol, BioXCell, BE0089
InVivoPlus polyclonal Syrian hamster IgG, BioXCell, BP0087

Validation

All the primary antibodies were validated by extensive previous studies and by the manufacturers.

1. https://www.merckmillipore.com/DE/en/product/Anti-APC-Ab-7-Mouse-mAb-CC-1,EMD_BIO-OP80
2. https://www.abcam.com/products/primary-antibodies/beta-2-microglobulin-antibody-ep2978y-ab75853.html
3. https://www.cellsignal.com/products/primary-antibodies/stat1-d1k9y-rabbit-mab/14994
4. https://www.biolegend.com/en-us/products/purified-anti-mouse-cd8a-antibody-157
5. https://labchem-wako.fujifilm.com/europe/product/detail/W01W0101-1974.html
6. https://www.sysy.com/product/234009#list
7. https://www.biolegend.com/en-us/products/purified-anti-beta-amyloid-1-11-antibody-13722
8. https://www.bio-techne.com/p/antibodies/mouse-serpin-a3n-antibody_af4709
9. https://www.merckmillipore.com/DE/en/product/Anti-Olig-2-Antibody,MM_NF-AB9610
10. https://www.rndsystems.com/products/mouse-pdgf-ralpha-antibody_af1062
11. https://www.thermofisher.com/antibody/product/MHC-Class-II-I-A-I-E-Antibody-clone-M5-114-15-2-Monoclonal/14-5321-82
12. https://www.scbt.com/p/isg15-antibody-f-9
13. https://www.abcam.com/products/primary-antibodies/myelin-basic-protein-antibody-12-ab7349.html
14. https://www.thermofisher.com/antibody/product/MBP-Antibody-Polyclonal/PA1-10008
15. ncbi.nlm.nih.gov/pmc/articles/PMC7116798/

16. https://www.merckmillipore.com/IN/en/product/Anti-Myelin-Associated-Glycoprotein-Antibody-clone-513,MM_NF-MAB1567

Secondary antibody:
1. https://www.thermofisher.com/antibody/product/Donkey-anti-Mouse-IgG-H-L-Highly-Cross-Adsorbed-Secondary-Antibody-Polyclonal/A-21202
2. https://www.thermofisher.com/antibody/product/Goat-anti-Mouse-IgG-H-L-Cross-Adsorbed-Secondary-Antibody-Polyclonal/A-21422
3. https://www.thermofisher.com/antibody/product/Goat-anti-Mouse-IgG-H-L-Cross-Adsorbed-Secondary-Antibody-Polyclonal/A-21235
4. https://www.thermofisher.com/antibody/product/Goat-anti-Rabbit-IgG-H-L-Cross-Adsorbed-Secondary-Antibody-Polyclonal/A-11008
5. https://www.thermofisher.com/antibody/product/Goat-anti-Rabbit-IgG-H-L-Cross-Adsorbed-Secondary-Antibody-Polyclonal/A-21428
6. https://www.thermofisher.com/antibody/product/Goat-anti-Rat-IgG-H-L-Cross-Adsorbed-Secondary-Antibody-Polyclonal/A-21434
7. https://www.thermofisher.com/antibody/product/Donkey-anti-Goat-IgG-H-L-Cross-Adsorbed-Secondary-Antibody-Polyclonal/A-21432
8.https://www.thermofisher.com/antibody/product/Donkey-anti-Goat-IgG-H-L-Highly-Cross-Adsorbed-Secondary-Antibody-Polyclonal/A32849
9. https://www.thermofisher.com/antibody/product/Donkey-anti-Rat-IgG-H-L-Highly-Cross-Adsorbed-Secondary-Antibody-Polyclonal/A-21208
10. https://www.thermofisher.com/antibody/product/Donkey-anti-Rabbit-IgG-H-L-Highly-Cross-Adsorbed-Secondary-Antibody-Polyclonal/A-31573
11. https://www.thermofisher.com/antibody/product/Donkey-anti-Goat-IgG-H-L-Highly-Cross-Adsorbed-Secondary-Antibody-Polyclonal/A32816

In vivo treatment:
https://bioxcell.com/invivoplus-anti-mouse-cd8a-bp0061
https://bioxcell.com/invivoplus-rat-igg2b-isotype-control-anti-keyhole-limpet-hemocyanin
https://bioxcell.com/invivoplus-anti-mouse-pd-1-cd279-bp0146
https://bioxcell.com/invivomab-anti-mouse-ctla-4-cd152-be0131
https://bioxcell.com/invivomab-rat-igg2a-isotype-control-anti-trinitrophenol-be0089
https://bioxcell.com/invivoplus-polyclonal-syrian-hamster-igg-bp0087

Animals and other organisms

Policy information about studies involving animals; ARRIVE guidelines recommended for reporting animal research

Laboratory animals	Mouse: C57BL/6J Janvier Labs N/A age 3,12,18,24 months<br>Mouse: B6.Cg-Tg(APPSwFlLon,PSEN1*M146L*L286V)6799Vas/Mmjax (5XFAD) age 4,6,10 months<br>Mouse: Apptm3.1Tcs (APP NL-G-F) age 12 months<br>Mouse: B6.129S7-Rag1tm1Mom/J (Rag1 KO) age 12 months from Jackson Laboratory
Wild animals	No wild animals were used in the study.
Field-collected samples	No field collected samples were used in the study.
Ethics oversight	All mouse experiments in this study were performed with the approval and according to the regulations of the District Government of Upper Bavaria and reported according to guidelines. CNS tissue was obtained from the archives of the Institute of Neuropathology at the University Medical Centre Goettingen. The study was approved by the local ethics committee (39/2/19)

Note that full information on the approval of the study protocol must also be provided in the manuscript.

Human research participants

Policy information about studies involving human research participants

Population characteristics	*Describe the covariate-relevant population characteristics of the human research participants (e.g. age, gender, genotypic information, past and current diagnosis and treatment categories). If you filled out the behavioural & social sciences study design questions and have nothing to add here, write "See above."*
Recruitment	*Describe how participants were recruited. Outline any potential self-selection bias or other biases that may be present and how these are likely to impact results.*
Ethics oversight	*Identify the organization(s) that approved the study protocol.*

Note that full information on the approval of the study protocol must also be provided in the manuscript.
