# Supplementary material for: Author Correction: T cell-mediated microglial activation triggers myelin pathology in a mouse model of amyloidosis
Source: Nat Neurosci. 2026 Oct 13;29(11):2890. doi: 10.1038/s41593-026-02502-x (PMC13724260; doi:10.1038/s41593-026-02502-x)
Supplement: Supplementary file 1 [file 41593_2026_2502_MOESM1_ESM.pdf]

nature neuroscience

Supplementary information

Author Correction: T cell-mediated microglial activation triggers myelin pathology in a mouse model of amyloidosis

In the format provided by the authors and unedited

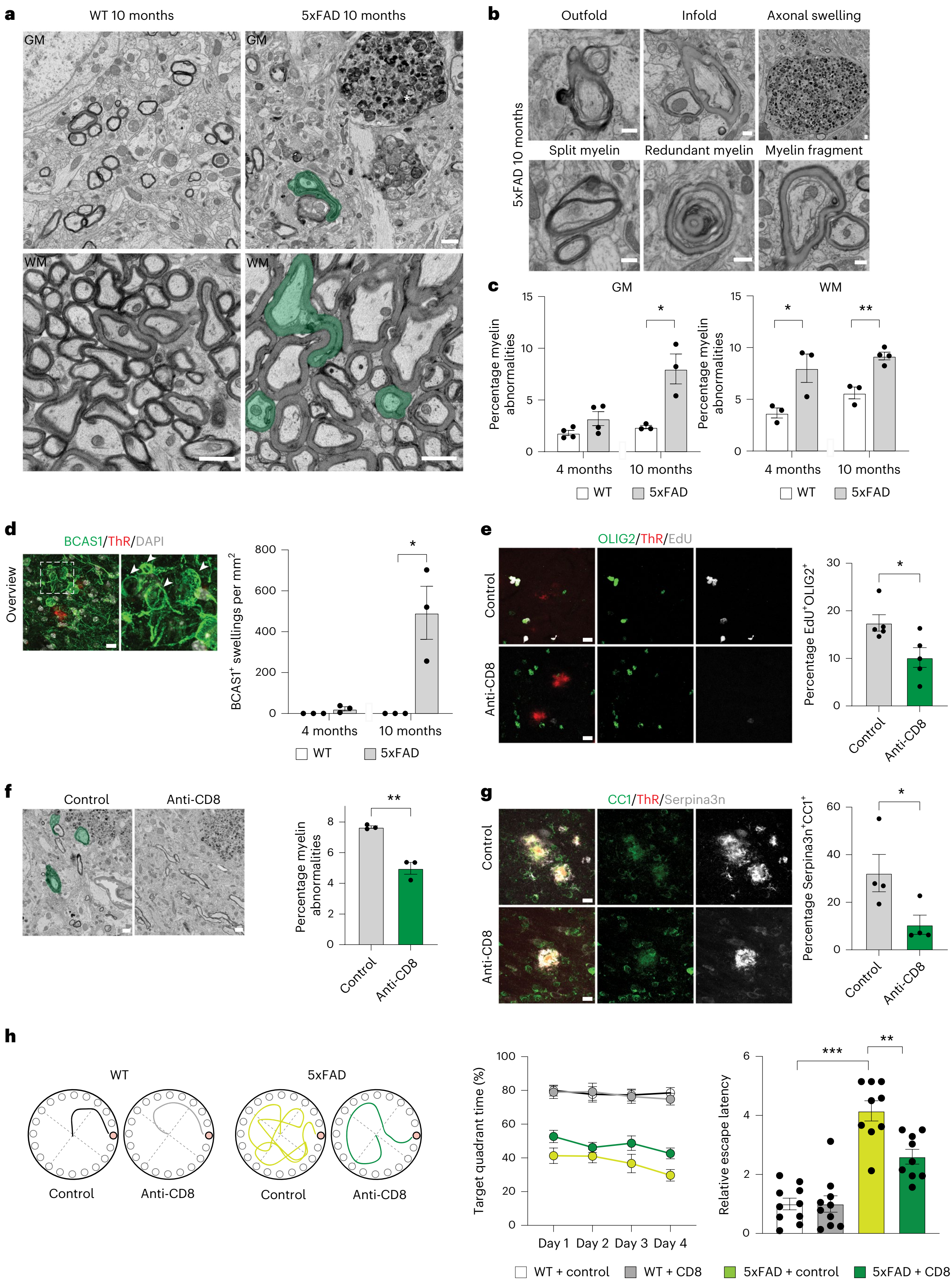

a
WT 10 months
5xFAD 10 months
GM
WM
b
Outfold
Infold
Axonal swelling
Split myelin
Redundant myelin
Myelin fragment
5xFAD 10 months
c
GM
WM
Percentage myelin abnormalities
4 months
10 months
WT
5xFAD
d
BCAS1/ThR/DAPI
Overview
BCAS1⁺ swellings per mm²
4 months
10 months
WT
5xFAD
e
OLIG2/ThR/EdU
Control
Anti-CD8
Percentage EdU⁺OLIG2⁺
f
Control
Anti-CD8
Percentage myelin abnormalities
g
CC1/ThR/Serpina3n
Percentage Serpina3n⁺CC1⁺
h
WT
5xFAD
Control
Anti-CD8
Target quadrant time (%)
Day 1
Day 2
Day 3
Day 4
Relative escape latency
WT + control
WT + CD8
5xFAD + control
5xFAD + CD8
